# Condensin ATPase motifs contribute differentially to the maintenance of chromosome morphology and genome stability

**DOI:** 10.1371/journal.pbio.2003980

**Published:** 2018-06-27

**Authors:** Roger Palou, Thillaivillalan Dhanaraman, Rim Marrakchi, Mirela Pascariu, Mike Tyers, Damien D’Amours

**Affiliations:** 1 Institute for Research in Immunology and Cancer, Département de Pathologie et Biologie Cellulaire, Université de Montréal, Montréal, Québec, Canada; 2 Ottawa Institute of Systems Biology, Department of Cellular and Molecular Medicine, University of Ottawa, Ottawa, Ontario, Canada; The Institute of Cancer Research, United Kingdom of Great Britain and Northern Ireland

## Abstract

Effective transfer of genetic information during cell division requires a major reorganization of chromosome structure. This process is triggered by condensin, a conserved pentameric ATPase essential for chromosome condensation. How condensin harnesses the energy of ATP hydrolysis to promote chromatin reorganization is unknown. To address this issue, we performed a genetic screen specifically focused on the ATPase domain of Smc4, a core subunit of condensin. Our screen identified mutational hotspots that impair condensin’s ability to condense chromosomes to various degrees. These mutations have distinct effects on viability, genome stability, and chromosome morphology, revealing unique thresholds for condensin enzymatic activity in the execution of its cellular functions. Biochemical analyses indicate that inactivation of Smc4 ATPase activity can result in cell lethality because it favors a specific configuration of condensin that locks ATP in the enzyme. Together, our results provide critical insights into the mechanism used by condensin to harness the energy of ATP hydrolysis for the compaction of chromatin.

## Author summary

In eukaryotes, the deletion of a single copy of most genes shows little or no detectable phenotype under standard proliferative conditions. This implies that a large reduction in the level of a gene product can be tolerated by eukaryotic organisms and that a “reserve capacity” is built in the protein machinery that drives most cellular processes. Here, we test if the main effector of chromosome condensation—the condensin complex—operates with a reserve enzymatic capacity in the execution of its multiple functions in vivo. To achieve this, we created an allelic series of mutations that selectively inactivate condensin ATPase activity in a graded manner. We show that many core functions of condensin can be maintained even at low levels of ATPase activity. Our data also reveal the existence of various thresholds of ATPase activity that are necessary and sufficient for the execution of different cellular functions by condensin. Notably, loss of genome stability at repetitive DNA is only observed when condensin ATPase activity is severely impaired. Taken together, our results reveal key insights into the process of ATP hydrolysis by condensin and how the energy it releases promotes genome remodeling and stability during cell division.

## Introduction

Chromosomes must undergo a major structural reorganization to allow the efficient and error-free segregation of genetic information during mitosis. This process is known as chromosome condensation and is promoted by the condensin complex [1–3]. The condensation of chromosomes has been described more than a century ago, and yet the molecular mechanism by which amorphous chromatin is reorganized into highly compact chromosomes is still poorly understood. The main effector of this reorganization is the condensin enzyme, a nuclear complex composed of two structural maintenance of chromosomes (SMC) proteins (Smc2 and Smc4), a kleisin subunit (Brn1/chromosome-associated protein H [CAP-H]), and two HEAT repeat–containing subunits (Ycg1/CAP-G and Ycs4/CAP-D) (reviewed in [4]). Condensin is a multifunctional enzyme that can bind and reanneal nucleic acids, as well as constrain knots and supercoils in the primary structure of DNA, which likely affect the topological configuration of chromosomes [5–8]. Importantly, the role of condensin during chromosome condensation depends largely on its mechanochemical properties and inherent ATPase activity.

The catalytic core of the condensin complex is formed by two SMC family subunits, a group of proteins involved in sister chromatid cohesion, DNA repair, and chromosome condensation (reviewed in [4,9]). SMC proteins are highly conserved in eukaryotes, and they are also found in other domains of life. The domain organization of this group of proteins is essential for function and is characterized by three globular domains (an N-terminal ATPase, a central hinge, and a C-terminal ATPase) separated by two alpha-helical segments [4]. To allow interactions between the terminal globular regions and create a functional ATPase “head domain,” individual SMC proteins fold back on themselves into a structure resembling a twisted hairpin, as suggested in early studies from the Earnshaw laboratory [10,11] and later confirmed by electron microscopy imaging [12]. Within condensin, interactions between the head domains of Smc2 and Smc4 create a bipartite ATPase unit that shares significant similarity with the extensively studied ATP-binding cassette (ABC) transporters [11]. In the case of ABC transporters, global structural rearrangements associated with the hydrolysis of ATP allow the translocation of small molecules through membranes. Despite the deep similarities between the ATPase domains of SMC proteins and ABC-type transporters, it has been difficult to extrapolate how condensin ATPase activity might promote chromosome compaction from our knowledge of the mode-of-action of ABC transporters.

The functional relevance of ATPase activity has also been studied in other SMC complexes, most notably in cohesin. This complex, composed of a catalytic core of Smc1 and Smc3 proteins, is required to hold the sister chromatids together during mitosis and meiosis [4]. In cohesin, ATP and its hydrolysis are necessary for DNA entrapment [13,14] and for tethering cohesin to chromosomes [15,16]. More recently, it has been shown that Smc3 can sense the presence of DNA and induce cohesin ring opening thanks to its ATPase activity [13]. In addition, Çamdere and colleagues have suggested that Smc1 and Smc3 ATPase head domains may work in a cooperative manner at different steps of the cohesion process [16]. The distinct molecular functions associated with different ATPase head domains could explain some of the asymmetry observed among SMC proteins within their cognate complexes, as previously observed with ABC transporters [17].

With respect to condensin, it is known that ATP hydrolysis plays an essential role in its function, as the abrogation of this activity leads to cell inviability [18,19]. Moreover, condensin activity is required even after chromosome assembly is apparently completed [20], suggesting that its ATPase activity must be continually active throughout mitosis to maintain chromosome structure. With respect to the mechanism of action, condensin binding to DNA is ATP independent [5,18], whereas its ability to constrain positive superhelical tension in double-stranded DNA (dsDNA) requires ATP [7]. Based on these observations, it has been suggested that ATP binding and hydrolysis may promote conformational changes in condensin that would allow DNA entrapment within its structure [19,21].

Ultimately, a key question for chromosome biology is how condensin uses the energy of ATP hydrolysis to convert amorphous chromatin into highly compacted chromosome in vivo. Since all ATPase-specific mutants tested so far in condensin are inviable (e.g., mutations in Walker A/B and C-motifs required for nucleotide binding and hydrolysis), it has been difficult to delineate the specific contribution of condensin ATP hydrolysis to the process of chromosome condensation. To address this important question, it is necessary to identify weakened or hypomorphic alleles of condensin that, despite being catalytically active, are defective enough to stabilize intermediate states in the process of chromosome compaction. To achieve this goal, we report here the characterization of an extensive collection of *smc4* mutants specifically defective in condensin ATPase activity.

## Results

### In silico modeling identifies Smc4 residues likely involved in ATP-dependent catalysis by condensin

**Fig 1:**
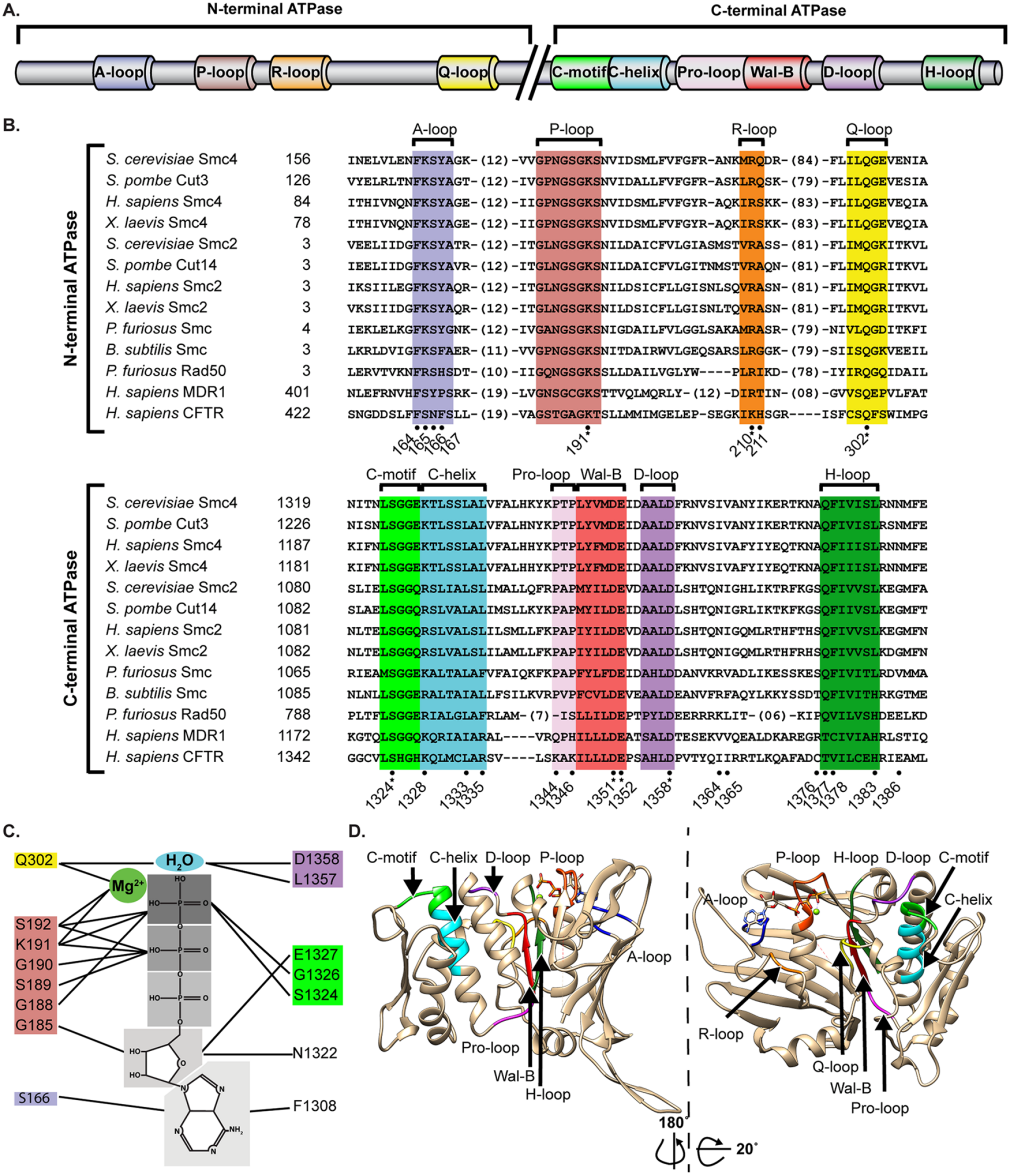
Structural alignment of the ATPase domains of ABC-type transporters and of the SMC components of the condensin complex. (A) Schematic representation of the motifs and loops of Smc4 ATPase domain. (B) The sequence of Smc2 and Smc4 subunits of *Homo sapiens*, *Xenopus laevis*, *Schizosaccharomyces pombe*, and *Saccharomyces cerevisiae* were aligned with SMC subunits from bacteria (*Bacillus subtillis*) and archaea (*Pyrococcus furiosus*) and two *H*. *sapiens* ABC-type transporters, MDR1 and CFTR. The names of the conserved loops and motifs of ABC-type transporters are marked above the sequences. Please note that the previously described DA-box is centered on the Walker B sequence and also encompasses the C-motif/helix, Pro-loop, and D-loop sections of SMC proteins [28]. (A dot symbol means novel mutations introduced in this study, whereas a star symbol signifies mutations previously described in condensin SMC subunits). (C) Major interactions predicted between the residues of Smc4 head domains and ATP prior to hydrolysis. The interactions are based on the X-ray crystallographic structure of MJ0796 (1L2T) and HlyB (1XEF) [29]. Note that C-motif and D-loop residues of Smc4 contact a second ATP molecule normally bound to Smc2. These contacts were depicted on the same ATP molecule in this schematic illustration for simplicity. (D) Bioinformatic model of the Smc4 ATPase domain using budding yeast cohesin subunit Smc1 (1W1W) [30] crystal structure as template. The major conserved motifs of ABC transporters are depicted. ABC, ATP-binding cassette; SMC, structural maintenance of chromosome.

The ABC-type ATPase domain is a characteristic feature of SMC proteins [11]. This family of ATPase domain is relatively uncommon in eukaryotes and is defined by the presence of 6 unique sequence motifs (A-, R-, Q-, D-, H-loops, and C-motif) in addition to the Walker A (P-loop) and Walker B consensus sequences found in most ATPases [22–26] (Fig 1A). Also conserved in ABC-type ATPase are the Pro-loop and C-helix regions adjacent to the Walker B and C-motifs, respectively [24,27]. The latter two regions have recently been connected to the hydrolysis of nucleotides and thus appear to be bona fide ATPase motifs. The ABC-type ATPase domain is most frequently found in membrane transporters, and an alignment of the amino acid sequence of typical human ABC transporters (MDR1 and CFTR1) with the ATPase domains of condensin SMC subunits reveals a strong evolutionary conservation of the ABC-specific sequence motifs in SMC proteins from various eukaryotes (Fig 1B). In fact, only the region encoding the H-loop and adjacent C-terminal sequence of the domain appears to diverge significantly in primary sequence among ABC-family ATPases (S1 Fig).

The role of most ABC-specific ATPase motifs has not been extensively characterized in SMC proteins. In contrast, the specific contribution of these motifs to the mode of action of ABC transporters is well documented (reviewed in [29]). These studies showed that the D-loop and the signature motif of one ATPase “head domain”/monomer cooperates with the active sites of the opposite ATPase head domain to allow the hydrolysis of ATP [31–34]. With respect to the role of each motif, the A-, P-, and H-loops contribute to the position and binding of ATP and cofactors required for catalysis, whereas the Walker B, D-loop, Q-loop, and C-motif are directly involved in ATP hydrolysis [29]. These analyses, together with the strong sequence conservation in ABC motifs, allowed us to predict the positions of Smc4 residues involved in ATP hydrolysis, as described schematically in Fig 1C.

To further validate the putative nucleotide–enzyme interactions, we also modeled Smc4 ATPase domain using crystallographic information from *S*. *cerevisiae* Smc1 (*ScS*mc1) (Fig 1D) [30,35]. *ScS*mc1 was used as a template because it shows substantial sequence identity to Smc4 (38% identity over their ATPase domains), and a high-resolution structure is available for its ATPase head domain. Overlap of known SMC structures from *Sc*Smc1 and *P*. *furiosus* Smc (*Pf*Smc) illustrates a high degree of conservation in structural organization within this family of proteins, even when primary sequence identity is weaker than that shared by Smc1 and Smc4 (i.e., 31% sequence identity in *Sc*Smc1 and *Pf*Smc ATPase head domain; S2 Fig). Importantly, our in silico model of Smc4 head domain points to a number of key residues that might interfere with ATP binding and/or hydrolysis when mutated (Fig 1D). Based on this information, we selected residues at 24 distinct positions within Smc4 ATPase domain for in-depth mutagenesis and phenotypic characterization (Fig 1B; positions marked with dots). These positions encompass all canonical ABC-type motifs as well as other residues that are predicted to be involved in catalysis. Of these, 18 correspond to residues that were not previously analyzed in SMC proteins (positions previously mutagenized are marked with a star in Fig 1B). We focused on residues that were highly conserved in each motif, as well as adjacent residues that are less conserved to avoid potential lethal mutations. We also mutated 2 regions not related to conserved ABC motifs, namely Ile1364-Val1365 and Gly1396, both of which are conserved in Smc4 but not in Smc2. Mutagenesis of Gly1396 was of particular interest because this residue is conserved in all eukaryotic Smc4s and prokaryotic SMCs but not in Smc2 nor ABC transporters (see alignment in S1 Fig). It was previously suggested that SMC proteins can be classified in two different subgroups based on sequence similarity between Smc1/4 and Smc2/3 at their most C-terminal sequences [36]. Mutation of Gly1396 was designed to test this notion and the functional importance of the more divergent sequences at the extreme C-terminus of Smc4 ATPase head domain (S1 Fig). Overall, this structural analysis provided a rationale for the creation of a total of 51 different mutations affecting Smc4 catalytic domain. We expected this collection of mutant alleles to cause defects of various severities in condensin ATPase activity, from mild to severe impairment in ATP hydrolysis in vivo.

### Targeted mutations in ABC-type motifs identify the H-loop and C-helix as mutational hotspots to generate thermosensitive alleles

**Fig 2:**
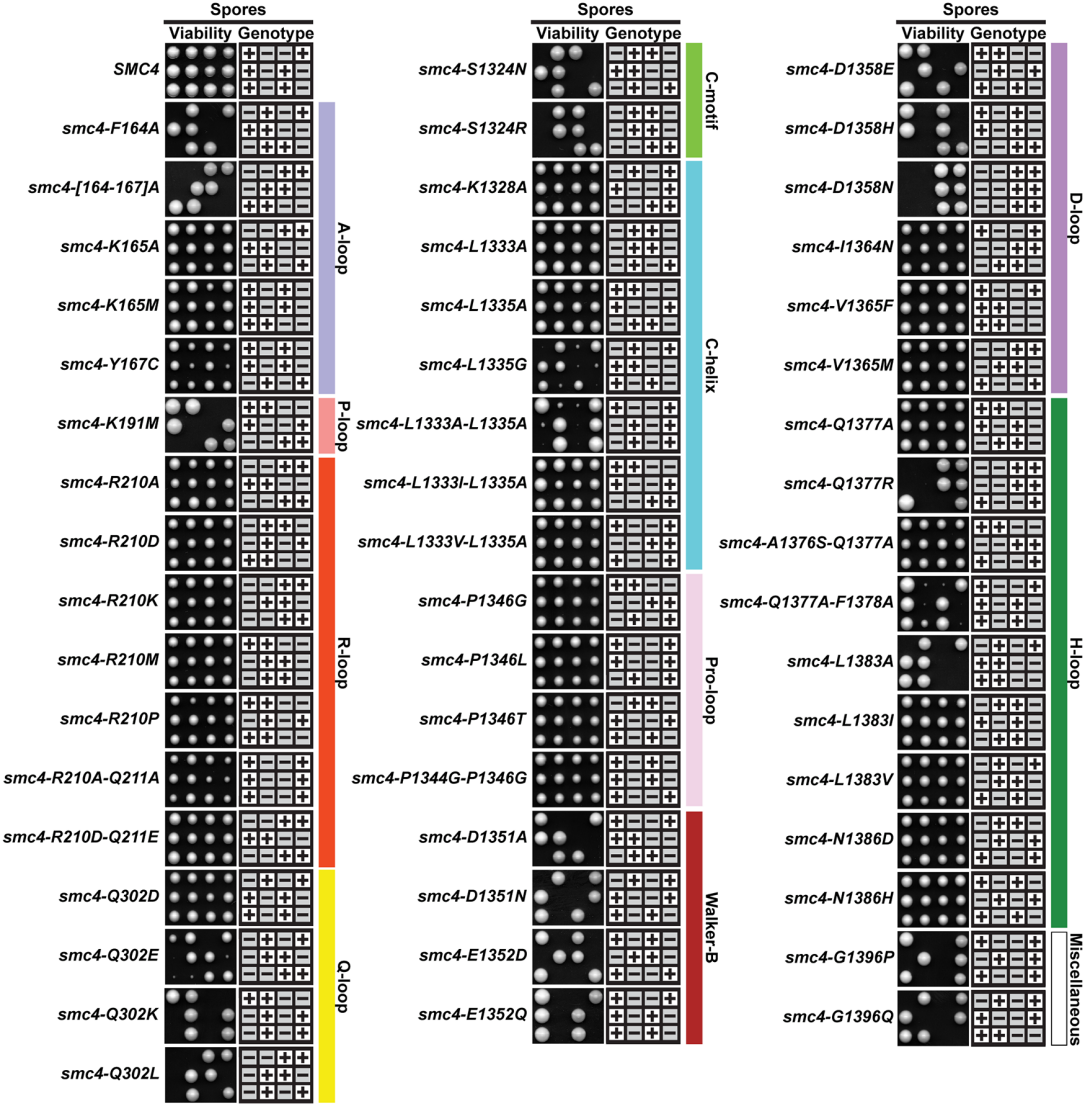
Identification of condensin ATPase residues crucial for cell viability. Heterozygous diploid strains carrying mutations in Smc4 ATPase domain were induced to sporulate, and the viability of the resulting haploid spores was determined after 2–4 days of growth on solid medium at 23 °C. Three typical tetrads of spores are shown per genotype. The genotype of spores was ascertained using the *HIS3MX6* marker associated with the mutant alleles of *SMC4* (i.e., minus sign means *smc4* mutant). To demonstrate the phenotype of lethal or very sick alleles of *SMC4* without ambiguity, tetrads containing these alleles were grown for longer periods of time (3–4 days) than fully viable tetrads (2–3 days).

**Fig 3:**
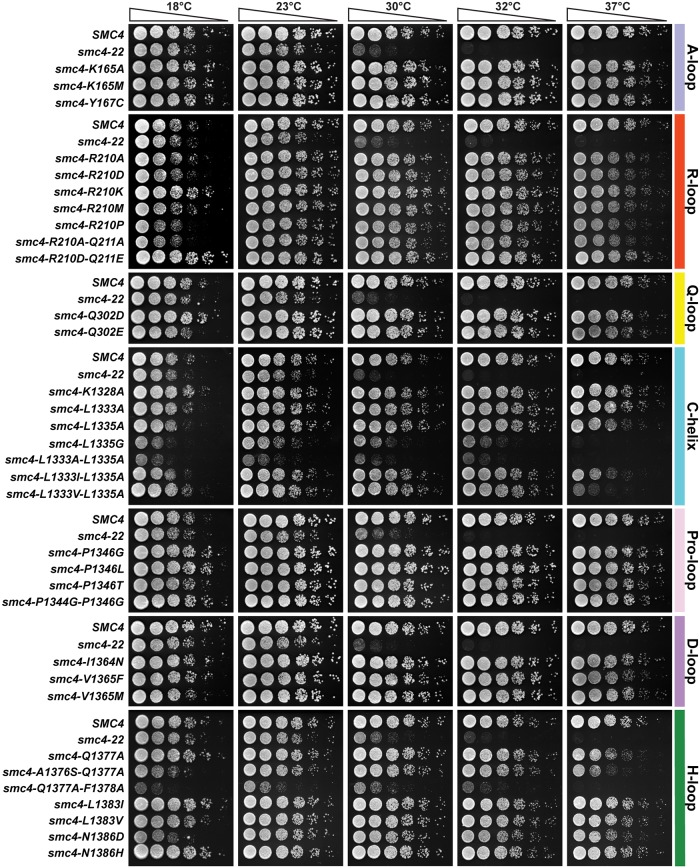
Characterization of the growth properties of viable Smc4 ATPase mutants. Five-fold dilution series of yeast cultures were spotted on solid rich medium and grown for 2–5 days at the indicated temperatures. Wild-type *SMC4* and *smc4-22* were used as positive and negative control, respectively. The results of this experiment are representative of those obtained in 3 independent repetitions. Note that plates carrying cold-resistant mutants at 18 °C were slightly undergrown relative to other plates in the series to allow the visualization of the enhanced growth phenotype at this temperature (i.e., compare wild-type strain growth in the R-loop and A-loop plates).

To facilitate the creation and analysis of a large collection of mutant alleles of *SMC4*, we used a gene replacement strategy in *S*. *cerevisiae*. Specifically, we constructed a series of heterozygous ATPase mutants in diploid cells and uncovered the phenotype associated with these *smc4* mutations after sporulation (Fig 2). Despite affecting residues highly conserved in ABC-type ATPases, most of the haploid mutants we generated were viable and showed normal growth at 23 °C. Only 18 out of the 51 mutants gave rise to tetrads with a 2:2 lethality phenotype cosegregating with *smc4* mutations (Fig 2). As expected, we isolated fully inactivating and/or lethal mutations in all the conserved motifs analyzed, except for the C-helix and R-loop (see Fig 3 for viable mutants). Much to our surprise, motifs proposed to be directly involved in ATP hydrolysis, such as the A-, R-, and Q-loops, could accommodate severe changes without strong effects. This tolerance was exemplified by Gln302 in the Q-loop, a position that tolerated mutation to negatively charged amino acids without detectable growth phenotype (i.e., *smc4-Q302E/D*; Fig 3). Likewise, introducing uncharged amino acids (Ala or Met) at the position of the critical arginine in the R-loop did not give rise to noticeable phenotypes. Only the charge-reversal substitution of Arg to Asp at position 210 in the R-loop resulted in a mild growth phenotype (Fig 3). This tolerance to mutation was in contrast to many other positions whose mutation gave rise to more consequential effects of viability. For instance, the position encoding Gly1396 at the extreme C-terminus of Smc4 was very sensitive to changes in amino acid (see lethality of *smc4-G1396P/Q* in Fig 2). Interestingly, this conserved position is unique to Smc4 and bacterial homologs of SMC proteins, and our result revealed the critical importance of this position for cell viability. Together, these results substantially expanded the known repertoire of structural motifs important for Smc4 ATPase activity and viability in budding yeast.

Next, we conducted a thorough analysis of the phenotype of viable mutants in our collection. First, we characterized the proliferative potential of viable Smc4 ATPase alleles using a 5-fold dilution series assay and monitored the ability of yeast to grow under progressively more challenging/suboptimal temperatures. From this collection of ATPase mutants, we identified 9 strains that showed thermosensitive growth behavior consistent with varying degrees of Smc4 inactivation (Fig 3). When compared to a known conditional allele, *smc4-22* [37], 3 ATPase mutants appeared to be acutely defective in Smc4 catalytic activity, as they showed severe proliferation defects even at optimal growth temperature (*smc4-L1335G; smc4-L1333A-L1335A* and *smc4-Q1377A-F1378A*). Several other mutants showed defects only at high temperature (37 °C), including strong (*smc4-L1333I-L1335A*; *smc4-L1333V-L1335A* and *smc4-A1376S-Q1377A*), intermediate (*smc4-L1335A* and *smc4-Q1377A*), and mild defects (most mutations affecting Smc4 R210 residue; Fig 3). We noticed that some mutants exhibited stronger proliferation defects after spore germination (Fig 2) when compared to normal vegetative growth conditions (*e*.*g*., *smc4-Q302E*; Fig 3). This likely reflects the fact that germination and normal vegetative growth are physiologically different states in yeast, and germination has unique genetic requirements relative to normal growth [38]. Among the viable mutants, we were unable to identify clear hypomorphic alleles affecting the Pro-loop and D-loop, despite the fact that these motifs are known to be important for ABC transporter and SMC protein functions [16,39]. Interestingly, all the thermosensitive mutations that showed intermediate or strong phenotypes are located in the C-terminal globular domain, in proximity to Leu1335 or Gln1377, key residues in the H-loop and C-helix motifs. A number of mutants in our ATPase collection also showed cold-sensitive growth at 18 °C, including *smc4-Q302E*, *smc4-A1376S-Q1377A*, and *smc4-N1386D*, but these phenotypes were relatively mild. Surprisingly, we found alleles that were cold-resistant, including all those containing mutations affecting the Pro-loop, as well as others scattered in various ABC motifs (Fig 3). Taken together, this structure-guided collection of mutations in the H-loop and C-helix motifs of Smc4 created many conditional alleles consistent with mutational hotspot regions in the protein. We then used this allelic series to explore the role of Smc4 ATPase activity in vivo.

### Conditional inactivation of Smc4 ATPase domain impairs chromosome condensation

**Fig 4:**
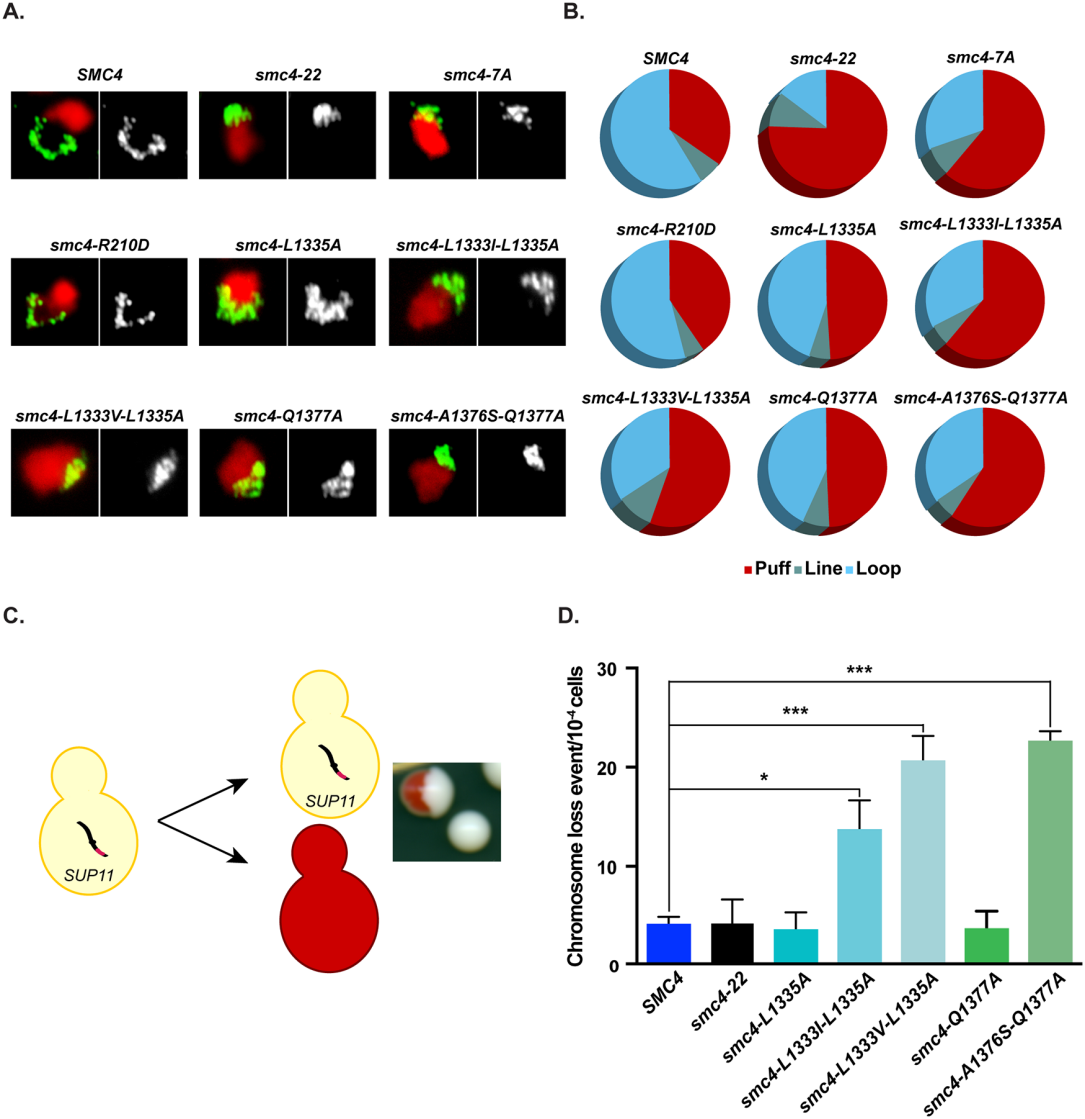
Smc4 ATPase mutants show condensation defects. (A) The morphology of the yeast rDNA locus was detected by FISH. Cells were grown asynchronously at 23 °C until exponential phase and shifted to 37 °C for 2 hours. Nocodazole was used to block cells in metaphase. Two conditional mutants, *smc4-22* and *smc4-*7A, were used as controls of strong and moderate condensation defects. Representative micrographs of the most abundant rDNA morphology observed for each mutant is shown. PI (red) and FITC (green) were used to label the nucleus and the rDNA locus, respectively. (B) Pie charts represent the quantification of each rDNA morphology. At least 100 nuclei were counted per mutant (*n* ≥ 3 for all the strains). See S1 Data for primary data. (C) Schematic representation of the CTF color colony assay. Colonies carrying CFIII are white because the *SUP11* gene on this chromosome suppresses the *ade2-1* red color phenotype. Loss of CFIII during cell division leads to the formation of red-sectored colonies (inset). (D) Quantification of the CTF phenotype in ATPase mutants of *SMC4*. Exponential cultures of yeast cells were grown at 23 °C and subsequently shifted to 37 °C for 180 minutes. Cells were then allowed to recover and grow at 23 °C on YPD medium without adenine supplementation. CFIII loss rate was calculated by dividing the number of half-sectored red colonies by the total number of colonies. *N* ≥ 3 for all yeast strains, and error bars represent SEM. The total number of colonies counted for each strain is reported in the Materials and methods section. Star symbols signify the following *p* values: * *p* < 0.05 and *** *p* < 0.001. See S1 Data for primary data. CFIII, chromosome III fragment; CTF, chromosome transmission fidelity; FISH, fluorescence in situ hybridization; FITC, fluorescein isothiocyanate; PI, propidium iodide; rDNA, ribosomal DNA; YPD, yeast extract peptone dextrose.

What is the contribution of Smc4 ATPase activity to chromosome condensation? Previous studies have shown that full inactivation of condensin ATPase activity is incompatible with cell viability [18]. However, it is not clear whether the lethality associated with these mutations is a consequence of chromosome condensation defects or defects affecting other essential processes carried out by condensin. To address this issue, we took advantage of the fact that thermosensitive alleles of condensin can be inactivated specifically in mitosis, allowing one to delineate the effect of Smc4 ATPase mutations on chromosome condensation without interference from other processes. To achieve this, we monitored the ability of conditional ATPase mutants (*smc4-R210D*; *smc4-L1335A*; *smc4-L1333I-L1335A*; *smc4-L1333V-L1335A*; *smc4-Q1377A*; and *smc4-A1376S-Q1377A*) to condense the ribosomal DNA (rDNA) locus in a nocodazole-induced early-mitotic arrest using fluorescence in situ hybridization (FISH). The phenotype of these cells was compared to that of wild-type *SMC4* and cells carrying *smc4-7A* and *smc4-22*, moderate and strong mutants of condensin, respectively [37]. The analysis of rDNA morphology by FISH is a sensitive approach to monitor chromosome condensation in *S*. *cerevisiae*, since the conformation of this locus changes dramatically during the cell cycle [40]. While wild-type *SMC4* cells synchronized in metaphase could condense DNA efficiently, as shown by the typical condensed “loop” configuration of the rDNA locus at 37 °C, *smc4-22* and *smc4-7A* presented uncondensed “puff” rDNA signal at restrictive temperature, as expected. With respect to the ATPase mutants, we observed condensation defects in all mutants except for the *smc4-R210D* allele (Fig 4A and 4B). The condensation-defective alleles can be classified into mild (*smc4-L1335A* and *smc4-Q1377A*) and moderate mutants (*smc4-L1333I-L1335A*; *smc4-L1333V-L1335A* and *smc4-A1376S-Q1377A*) according to the fraction of cells that exhibited uncondensed “puff” rDNA morphology (i.e., less or more than a threshold of 50% uncondensed rDNA; Fig 4A and 4B and S1 Table). However, none of the Smc4 ATPase alleles were as defective in rDNA loop formation as *smc4-22* (i.e., 75.7% “puff” phenotype), which was expected because their proliferation defect was weaker than *smc4-22* at nonpermissive temperature (Fig 3). These results, together with the cell proliferation assay, highlight the existence of a clear correlation between the severity of the thermosensitive phenotype and the condensation defects in *smc4* ATPase mutants.

To investigate the biological relevance and contribution of the ATPase activity of condensin to genome stability, we conducted experiments to assess chromosome segregation fidelity in *smc4* mutants. We took advantage of a nonessential chromosome that carries a dominant suppressor (*SUP11*) as an assay to monitor chromosome loss during cell division. Specifically, cells that efficiently segregate this chromosome during mitosis maintain a white colony color due to the suppression of the *ade2-1* allele in the strain background, whereas cells that lose the chromosome generate red pigments/sectors in yeast colonies [41] (see inset in Fig 4C). As expected, many of the ATPase-deficient strains of *smc4* showed significant increases in red sector formation/chromosome loss during cell proliferation (Fig 4D). In particular, mutants that showed the strongest defects in chromosome condensation—such as *smc4-L1333I-L1335A*, *smc4-L1333V-L1335A*, and *smc4-A1376S-Q1377A*—were also the most defective in the fidelity of chromosome segregation during mitosis. These results demonstrated that the ultimate biological consequence associated with the impairment of condensin ATPase activity is an inability to effectively segregate chromosomes during cell division.

**Fig 5:**
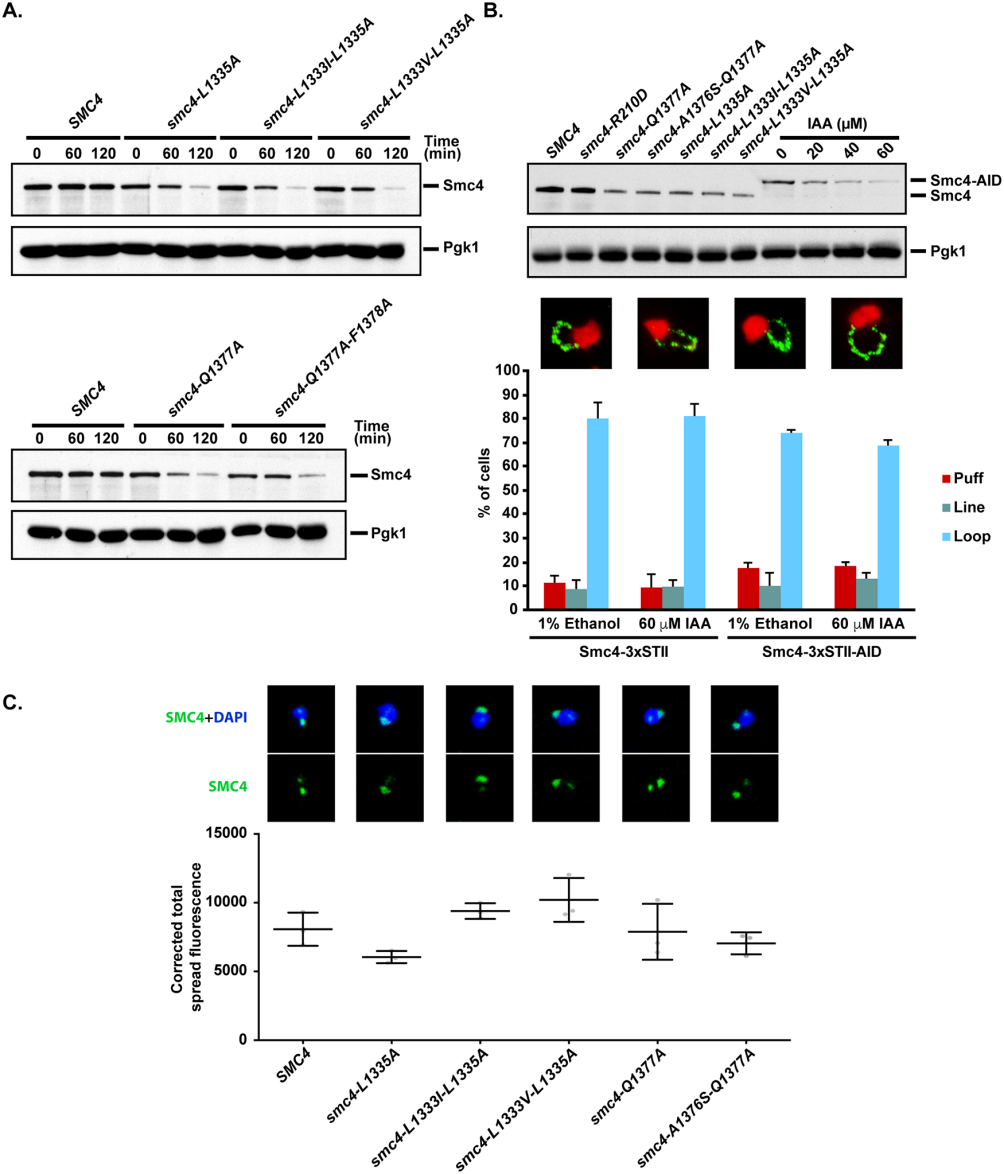
The phenotype of ATPase-defective mutants of Smc4 is not solely due to changes in protein abundance or localization to chromatin spreads. (A) Cells were grown asynchronously at 23 °C until exponential phase, and then exposed to 37 °C for 2 hours. Cells were collected every 60 minutes and processed for immunoblot analysis. Pgk1 serves as a loading control. Smc4 abundance was evaluated by immunoblot analysis using 3xStrepTagII epitopes at the C terminus of the protein. (B) Reduction of Smc4-AID protein abundance by auxin/IAA do not impact chromosome condensation. Upper panels: Regulation of Smc4-AID levels. Cells were grown asynchronously at 23 °C until exponential phase and exposed to different concentrations of auxin for 2 hours at 37 °C. Wild-type and Smc4 ATPase mutants’ protein levels were included as reference. Lower panels: Chromosome condensation at low Smc4 levels. Cells were grown asynchronously at 23 °C until exponential phase and shifted to 37 °C for 2 hours. Nocodazole was used to block cells in metaphase, and 60 μM auxin/IAA was used to reduce Smc4 levels. Nuclei and rDNA were stained with PI (red) and FITC (green), respectively. For each condition, a representative micrograph of the most prominent rDNA phenotype by FISH is shown. At least 100 nuclei were counted per condition (*n* = 3 for all the strains). Error bars represent SD. See S1 Data for primary data. (C) Mitotic cells synchronized in metaphase with nocodazole were spheroplasted and processed for chromatin spread analysis as previously described [43]. Images on the top show typical chromatin spreads prepared from various ATPase mutants of Smc4, whereas the bar graph under the spread pictures shows a quantification of Smc4-Myc fluorescence intensity on chromatin. The various yeast mutants showed no significant differences in condensin binding to chromatin spreads. See S1 Data for primary data. Nuclei were labeled with DAPI (blue), and Smc4 was labeled with FITC (green). At least 100 spreads were analyzed for each condition (*N* = 3). Error bars represent SD. AID, auxin inducible degron; DAPI, 4’,6-diamidino-2-phenylindole; FISH, fluorescence in situ hybridization; FITC, fluorescein isothiocyanate; IAA, indole-3-acetic acid; PI, propidium iodide; rDNA, ribosomal DNA.

Since condensin complex formation is essential for viability [42], we wished to investigate whether the defects we observed in cells expressing ATPase mutants of Smc4 were caused by a loss of ATPase activity per se or were a reflection of indirect effects on protein stability and/or complex formation in vivo. Purification of the ATPase mutants of Smc4 showed that they assemble with all other subunits of the enzyme into condensin holocomplexes (see S4 Fig below). In contrast, immunoblot analysis revealed that many of the *smc4* ATPase alleles exhibited reduced protein levels compared to wild-type Smc4 when cells were shifted from 23 °C to 37 °C (Fig 5A). This result prompted us to assess whether Smc4 levels might underpin the chromosome condensation defect of condensin ATPase mutants. To address this issue, we down-regulated wild-type Smc4 abundance using an auxin inducible degron (AID) allele (i.e., Smc4-AID) and monitored chromosome condensation in those cells. When Smc4 levels were reduced to the same extent as that of the most severe ATPase mutants we created, we did not detect any defect in chromosome condensation (i.e., no increase in the rDNA “puff” phenotype; Fig 5B). Taken together, these results indicate that the condensation defect observed in ATPase mutants is not the sole consequence of reduced Smc4 protein levels at restrictive temperature and instead reflects an additional loss of function in ATPase activity. These results are consistent with the previous observation that cells express an excess of Smc4 protein under normal conditions and that reduction of Smc4 levels to under 10% wild-type levels does not affect cell proliferation [37]. Finally, we investigated if the localization of ATPase mutants of condensin was altered in cells. Analysis of Smc4 signals on chromatin spreads revealed that all but one ATPase mutant of Smc4 were properly localized on yeast chromosomes (Fig 5C). Specifically, only the Smc4-L1335A mutant appeared to be present at lower levels on chromatin spread relative to wild-type Smc4. Taken together, these results indicated that the phenotype of ATPase-defective mutants of Smc4 was not a consequence of condensin mislocalization in vivo.

### Severe inactivation of Smc4 ATPase domain leads to a contraction of the rDNA array

**Fig 6:**
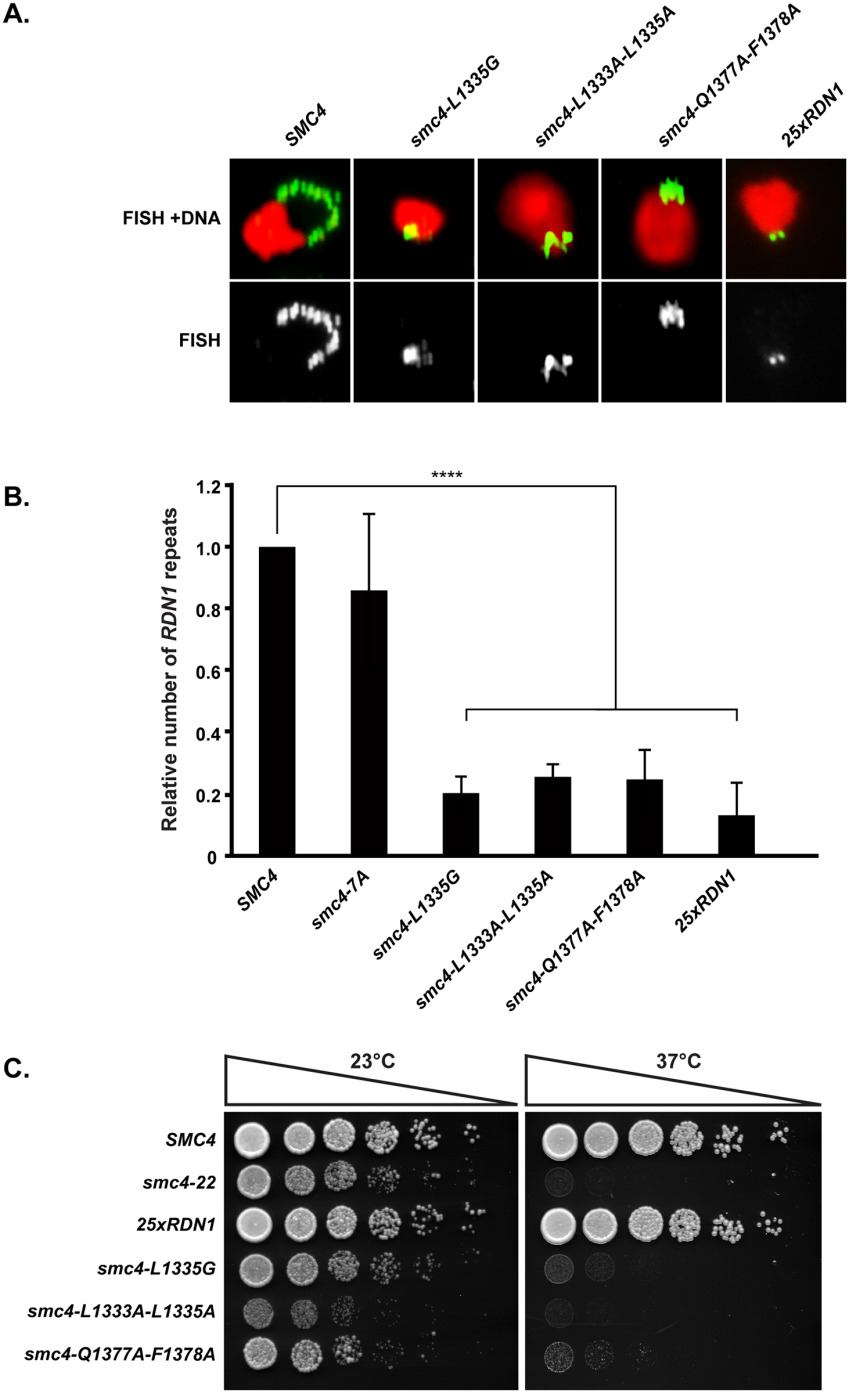
Three *smc4* ATPase mutants show rDNA array contraction. (A) Chromosome morphology was detected by FISH. Cells were grown asynchronously at 23 °C until exponential phase and shifted to 37 °C for 2 hours. Nocodazole was used to block cells in metaphase. Nuclei and rDNA were stained with PI (red) and FITC (green), respectively. For each condition, a representative micrograph of the most prominent rDNA phenotype is shown. (B) Representation of the relative amount of *RDN1* repeats in Smc4 ATPase mutants compared to a wild-type strain. *RDN1* copy number was determined by quantitative PCR analysis and standardized using *SMC4* as a single copy gene. Error bars represent SD, and n.s. indicates no significant difference. See S1 Data for primary data. (C) Five-fold dilution series of yeast cultures were spotted on plates containing rich medium and grown for 2 days at 23 °C and 37 °C. Wild-type *SMC4* or *smc4-22* were used as positive and negative control, respectively. The results of this experiment are representative of those obtained in 3 independent repetitions. FISH, fluorescence in situ hybridization; FITC, fluorescein isothiocyanate; PI, propidium iodide; rDNA, ribosomal DNA.

Among the collection of Smc4 ATPase mutants we isolated, a subgroup showed very severe growth defects, even at permissive temperature, and were completely inviable at 37 °C (i.e., *smc4-L1335G*, *smc4-L1333A-L1335A*, and *smc4-Q1377A-F1378A*; Fig 3). Analysis of rDNA morphology in these three ATPase mutants revealed an rDNA FISH signal that was significantly less intense than that of wild-type cells under the same conditions (Fig 6A). The probe we used to detect the budding yeast rDNA locus recognizes the 100–200 repeats of a 9.1 kb sequence (*RDN1*) carried on chromosome XII [44]. Since FISH signal at this locus directly correlates with rDNA copy number [45], one explanation for the weaker rDNA signal we detected in ATPase mutants might be that they have lost some of their rDNA repeats. Previous work has shown that condensin interacts directly with the rDNA locus [46] and that its activity is necessary to maintain copy number stability in the rDNA array [45]. In light of this, we examined whether severe mutations in Smc4 ATPase domain led to rDNA array contraction at permissive temperature. We used quantitative PCR to assess the number of rDNA repeats in mutant strains relative to wild-type cells. As shown in Fig 6B, the three *smc4* mutants with the lowest FISH signal exhibited a reduction of more than 70% in rDNA copy number when compared to wild-type cells. Consistent with this estimation, the ATPase mutants also contained more *RDN1* copies than a control strain that was engineered to carry approximately 25 copies of the rDNA repeat [47]. We confirmed that a strain carrying approximately 25 copies of *RDN1* has normal kinetics of cell cycle progression, as previously published [47], and showed that the proliferation capacity of our three ATPase mutants is significantly impaired even at permissive temperature (Fig 6C). These results indicate that strains expressing ATPase-defective Smc4 experience additional defects in chromosome morphology and/or stability. These defects may act separately or in a synthetic manner with the rDNA array contraction to cause slow growth at a permissive temperature.

### Phenotype of condensin mutants carrying an “ATPase-dead” mutation

**Fig 7:**
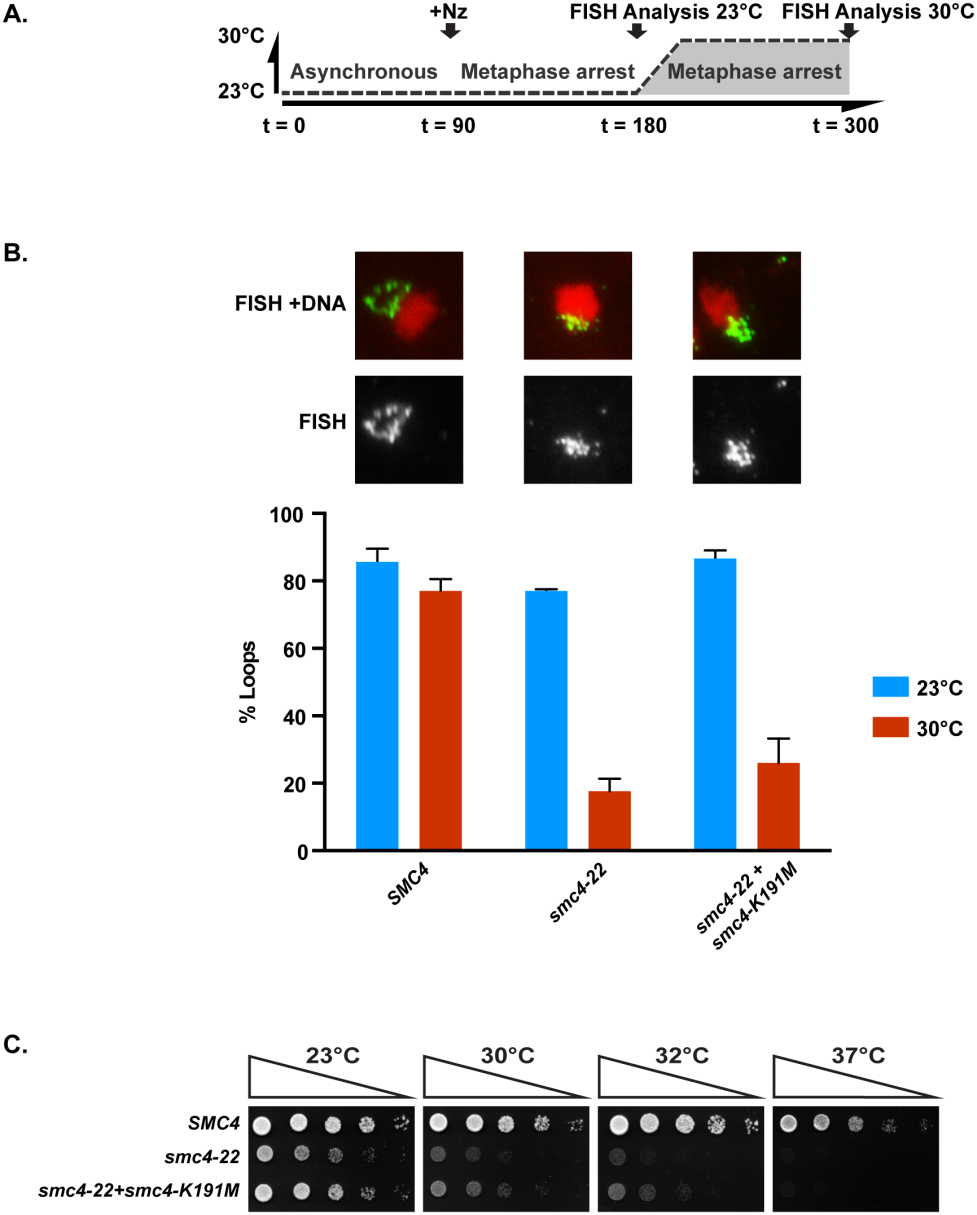
Full inactivation of Smc4 ATPase abrogates rDNA condensation and impairs cell growth. (A) Schematic representation of the experimental conditions used to monitor chromosome condensation in the presence of an ATPase-dead mutant of Smc4. (B) rDNA morphology analysis in cells expressing *SMC4*, *smc4-22*, and *smc4-22* with *smc4-K191M*. Micrographs showing representative rDNA morphologies in *smc4* mutants at 30 °C are shown on top, whereas the quantification of the rDNA loop morphology is shown below. rDNA FISH analysis was carried out as before, and at least 100 cells were counted for each experimental condition (*n* = 3; error bars represent SEM). See S1 Data for primary data. (C) Viability of cells expressing an ATPase-dead mutant of condensin. Cells were diluted 5-fold on solid medium and grown at the indicated temperatures for 2–3 days. FISH, fluorescence in situ hybridization; rDNA, ribosomal DNA.

The conditional lethal mutants identified above are viable at permissive temperature, suggesting that the encoded proteins are not completely defective in condensin ATPase activity. It is also conceivable that some enzymatic activity is retained at nonpermissive temperature or that the mutant proteins act as dominant negatives, thus hiding the true phenotype associated with complete loss of ATPase function in condensin complexes. To reveal the full phenotype associated with abrogation of condensin enzymatic activity, we expressed an “ATPase-dead” allele of *SMC4*, *smc4-K191M*, in the background of a strong temperature-sensitive mutant of the same gene (*smc4-22*; inviable at approximately 30–32 °C; Fig 3). An analogous mutation in other SMC proteins is known to fully abrogate ATP hydrolysis in vitro [19,39]. The *smc4-22* mutant was allowed to establish chromosome condensation at permissive temperature (23 °C) and was subsequently inactivated by shifting to 30 °C (Fig 7A). This experimental regimen impaired rDNA condensation after the temperature shift in the *smc4-22* mutant but not in cells expressing wild-type *SMC4* (Fig 7B). In contrast, expression of the ATPase-dead *smc4-K191M* allele in this context did not alleviate the condensation defect and inviability of the *smc4-22* mutant at 30 °C (Fig 7B and 7C). We did notice a very slight suppression of the cell growth defect of *smc4-22* mutants when expressing *smc4-K191M* (Fig 7C), perhaps reflecting a degree of interallelic complementation between *smc4* alleles, as previously reported for mutations affecting the cohesin complex [48]. Together, our experiments demonstrate that condensin ATPase activity is essential for effective chromosome condensation and maintenance of cell viability.

### Conditional mutants in the H-loop and C-helix of Smc4 specifically affect ATP hydrolysis

**Fig 8:**
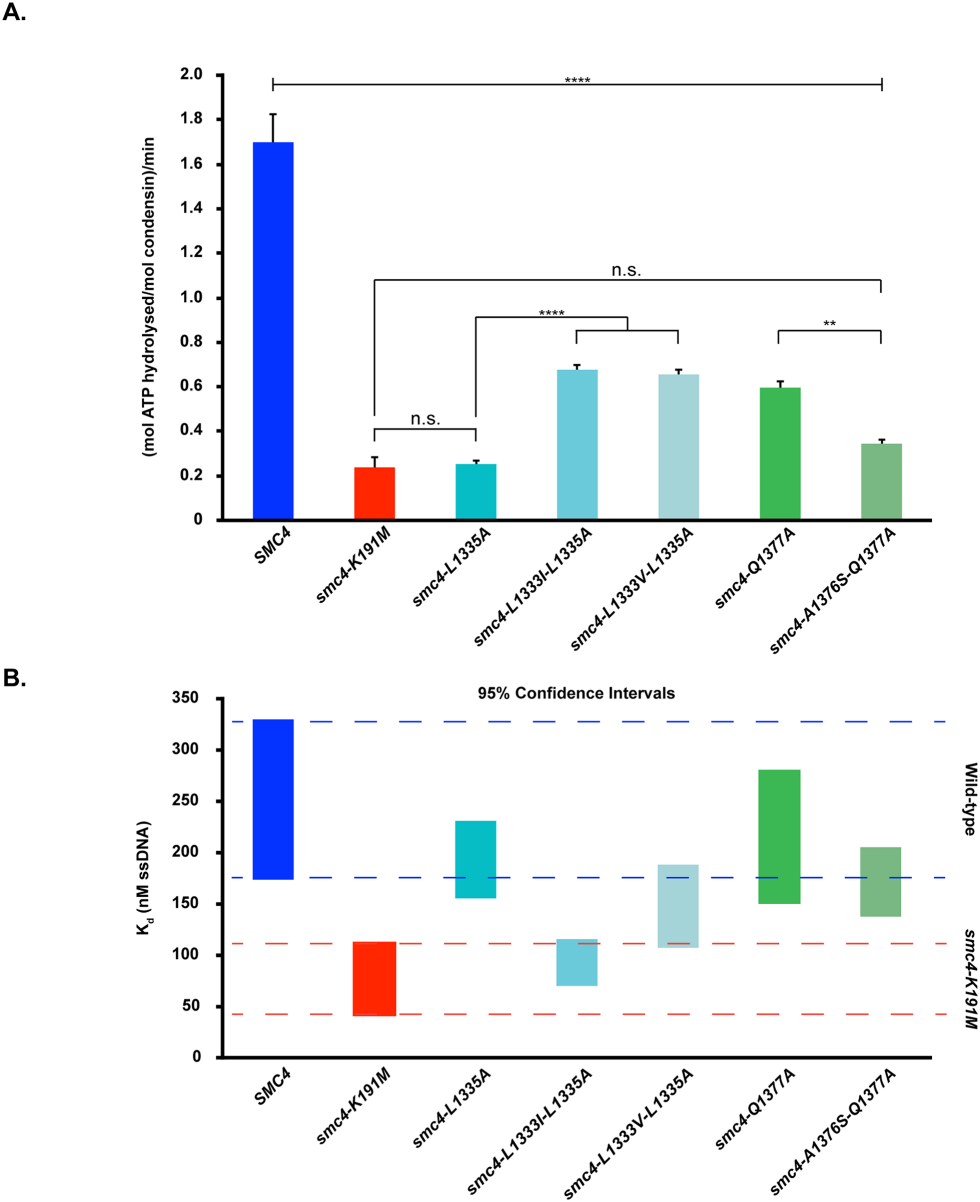
Smc4 ATPase mutations that compromise chromosome condensation exhibit reduced ATP hydrolysis in vitro. (A) ATPase activity of Smc4, Smc4-K191M, and the H-loop and C-helix mutants were compared using equal amounts of protein (1.5 μmol). Each condensin complex was incubated for 1 hour in ATPase buffer at 30 °C. ADP accumulation was measured with ADP-Glo luminescence kit (Promega). Error bars represent SEM (*N* ≥ 8 for all complexes). Star symbols signify the following *p* values: ** *p* < 0.01 and **** *p* < 0.0001. N.s. indicates no significant difference. (B) DNA binding to 60-nt ssDNA of condensin complexes used in (A) was analyzed using fluorescence anisotropy. Increasing amounts of each condensin complex were incubated with 10 nM 6-FAM ssDNA until the signal became saturated. The binding *K*_*d*_ was calculated using One site-Specific binding with Hill slope equation, and the 95% confidence interval for each complex is depicted in the figure (*N* = 3 for all complexes). See S1 Data for primary data. 6-FAM, 6-carboxyfluorescein; ssDNA, single-stranded DNA.

We next wished to determine whether the phenotype of the ATPase mutants that we created could be attributed to impaired ATPase activity in condensin. To achieve this, we purified condensin complexes containing wild-type Smc4, the 5 ATPase mutants showing significant condensation defects and, as a negative control, a previously described ATPase-dead mutant, Smc4-K191M [19,39]. We used a rapid overexpression approach to purify these complexes from yeast in order to bypass low viability issues associated with the expression of ATPase-defective alleles of *SMC4* [49]. We then assayed the ATPase activity of these complexes using a luminescence assay that monitors production of ADP upon ATP hydrolysis. In this assay, wild-type condensin hydrolyzed 1.69 mol of ATP per mol of enzyme per minute, which is very similar to the value previously reported for the holocomplex using radioactive ATP [50]. On the other hand, the ATPase-dead control (Smc4-K191M) was severely reduced for ATPase activity (0.24 mol ATP hydrolyzed per mol of condensin per minute). When we compared ATPase motif mutants of condensin with controls, all exhibited far less ATP hydrolysis than the wild-type complex, and the more severe mutants exhibited enzymatic activity similar to that of condensin complexes containing Smc4-K191M (Fig 8A). Interestingly, while a general correlation was observed between the extent of ATPase dysfunction and the severity of the condensation defects in H-loop mutants, no such correlation was observed with the C-helix mutants. Specifically, complexes containing Smc4-L1335A and Smc4-A1376S-Q1377A mutants showed ATPase activity comparable to that of the ATPase-dead control while maintaining cell viability (Figs 3 and 8A). One possible explanation for this result is that different mutations in the C-helix of Smc4 acted as separation-of-function alleles or, alternatively, that the C-helix mutants had a minimal threshold of ATPase activity required to maintain cell viability (whereas Smc4-K191M did not meet this threshold). It is also possible that the Smc4-K191M ATPase-dead allele gained a novel dominant activity that impaired cell viability independently of its catalytic defect per se.

To explore the nature of this conundrum, we used a UV cross-linking approach [19] to monitor the ATP-binding capacity of condensin complexes containing normal (Smc4), moderate (Smc4-Q1377A), and low (Smc4-L1335A) ATPase activity. This approach revealed that Smc2 and Smc4 bound equally well to radiolabeled ATP in wild-type condensin, as expected (S3 Fig; long exposure). In contrast, complexes containing Smc4-L1335A and Smc4-Q1377A showed a marked reduction in Smc4 nucleotide binding compared to Smc2, consistent with their reduced ATPase activity. Surprisingly, Smc2 showed a large—and somewhat paradoxical—increase in ATP retention in condensin complexes containing the ATPase-dead mutant Smc4-K191M (S3 Fig; short exposure). This result suggested that the lethality of *smc4-K191M* allele could be due to condensin complexes being dominantly and/or constitutively locked in a deleterious conformation that traps ATP in Smc2. This interpretation would be in line with the fact that yeast carrying the *smc4-L1335A* allele were viable (Fig 3), whereas those carrying *smc4-K191M* were not (Fig 2), despite both mutants showing comparable ATP hydrolysis defects (Fig 8A). This result raised the possibility that the ATPase activity of condensin may impact or otherwise regulate other biochemical activities of the complex. One activity that is likely to be regulated by ATP hydrolysis is the ability of condensin to bind DNA and chromosomes. To test this possibility, we conducted fluorescence anisotropy experiments to monitor the affinity of condensin complexes for short DNA substrates. First, with respect to the control complexes, we observed that both the wild-type condensin and the ATPase-dead control (Smc4-K191M) were able to bind 60-nt single-stranded DNA (ssDNA) (Fig 8B). However, the affinity of the ATPase-dead control for short ssDNA was significantly higher (*K*_*d*_ ≈ 76 nM) than that of the wild-type complex (*K*_*d*_ ≈ 251 nM) (Fig 8B and S4 Fig). Likewise, when we analyzed the DNA-binding capacity of condensin complexes containing our ATPase mutants, we observed that all mutant proteins were able to bind DNA effectively. Moreover, all ATPase mutants showed an affinity for DNA that was slightly higher (Smc4-L1335A, *K*_*d*_ ≈ 193 nM; Smc4-L1333V-L1335*A*, *K*_*d*_ ≈ 147 nM; Smc4-Q1377A, *K*_*d*_ ≈ 215 nM; Smc4-A1376S-Q1377A, *K*_*d*_ ≈ 171 nM) or much higher (Smc4-L1333I-L1335A, *K*_*d*_ ≈ 92 nM) than that shown by wild-type condensin (Fig 8B and S4 Fig). Since all Smc4 mutants tested could bind to DNA, we next asked whether the binding was reversible. To monitor this property, we conducted competition experiments in which condensin complexes prebound to labeled ssDNA were incubated in the presence of an excess of nonlabeled ssDNA. Under this condition, all condensin complexes were able to release prebound-labeled ssDNA (S4B Fig). Interestingly, condensin complexes containing the Smc4-K191M exchanged ssDNA at lower concentration than the wild-type complex, consistent with the affinity displayed in binding assays. Taken together, our biochemical analyses indicated that the mutations we introduced in the H-loop and C-helix of Smc4 decreased the rate of condensin ATP hydrolysis without compromising the ability to bind DNA tightly. Importantly, the effects we observed were not due to major changes in subunit stoichiometry in the mutant complexes (S4C Fig).

## Discussion

Condensin was first described as an ATPase required for the organization of chromatin [1–3,10]. After its discovery, it was shown that condensin ATP hydrolysis activity is essential, since mutation of key residues in canonical ATPase motifs of the SMC subunits led to cell death [reviewed in 51]. More recent in vitro studies have shown that condensin is necessary for both establishing and maintaining the condensation state of mitotic chromosomes [20]. However, it is still unknown how the complex uses the energy released during nucleotide hydrolysis to reconfigure chromosome morphology in vivo. To understand this fundamental question, it is important to identify condensin mutants that compromise ATP hydrolysis while preserving enough activity to sustain cell viability and allow visualization of the consequences associated with impaired ATPase activity. In this study, we have generated an extensive allelic series specifically targeting Smc4 ATPase motifs to explore the role of ATP hydrolysis in condensin function. Our results show, for the first time, that it is possible to significantly reduce the ATPase activity of condensin while maintaining cell viability. Moreover, our findings reveal the existence of unique thresholds for condensin activity in the execution of its diverse functions in cells (S2 Table).

### Revealing the contributions of ABC-type ATPase motifs in chromosome morphogenesis

SMC complexes have two defining structural characteristics, a ring-like architecture and the presence of a bipartite ATPase domain of the ABC family. In this study, we show that mutations in most of the ABC-type ATPase motifs of Smc4—with the notable exception of the R-loop and C-helix—can severely compromise budding yeast viability, thereby demonstrating their relevance to the mechanism of ATP hydrolysis by condensin. Interestingly, our results also show that in spite of the highly conserved nature of the ABC-type motifs in Smc4 ATPase domain, the importance of specific conserved residues for nucleotide catalysis does not appear to be equivalent in all cases. For instance, the D-loop and its characteristic Asp1358 residue represent a good example of conservation in both structure and function. This position has been shown to form the ATP hydrolysis site in ABC transporters and also in cohesin SMC subunits [16,52]. We observed that mutation of this residue in Smc4 had dramatic consequence for yeast viability. On the other hand, the conserved Arg210 in the R-loop is an example of structural but not functional conservation. This position has been implicated in the stimulation of ATP hydrolysis by DNA in *Pf*Smc and Rad50 complexes [23,32]. However, introducing multiple different residues at this position in yeast Smc4 did not significantly decrease cell viability. Since the ATPase activity of condensin is also enhanced in the presence of DNA, we envision that the stimulatory effect of DNA is mediated by a different region/motif in the protein. An alternative explanation for the lack of effect of specific mutations could be that Smc4 is structurally resilient and adaptable, thereby buffering protein activity from otherwise deleterious mutations. It is also possible that evolutionary specialization in the function and mode of action of SMC proteins leads to different specific roles for the ABC-type motifs in their cognate complexes. For instance, while *Pf*Smc R-loop is critical for ATPase activation in the presence of DNA (14-fold stimulation) [23], the stimulation of yeast condensin ATPase by DNA is more modest (4-fold stimulation) [5,50,53]. The fact that some ATPase motifs appear to be very resilient to mutations (e.g., the previously mentioned R-loop or the Pro-loop), whereas others are highly sensitive to mutagenesis (e.g., the Walker B or the C-motif), may reflect the catalytic mechanism of the enzyme. For instance, the capacity of some ATPase motifs to tolerate mutations may indicate that they are not intimately involved in catalysis per se, but they may instead fulfill ancillary or supporting roles in catalysis (i.e., analogous to the C- and R-spines in kinase domains; [54]). An unexpected group of yeast mutants identified in our allelic series are those that confer cold-resistant growth properties. This type of allele is rarely reported, partly because cellular proliferation at cold temperatures is not frequently tested, and the molecular determinants that lead to this type of phenotype are still poorly understood. It is conceivable that cold-resistant mutations give rise to faster growth kinetics by stimulating enzyme catalysis or stabilizing protein–protein interactions via hydrophobic interfaces, two processes that are negatively impacted by lower temperatures. The molecular basis of cold-resistant growth in condensin mutants remains to be determined.

The main goal of our structure-guided genetic analysis was to investigate for the first time the relative importance and contribution of ABC-type ATPase motifs to the mode-of-action and cellular functions of condensin. Importantly, our study identified 5 new residues in Smc4 ATPase head domain that play important roles in the establishment of chromosome morphology. These residues are located in 2 motifs of the SMC ATPase domain that have been poorly studied and show a remarkable resilience to mutation, namely the C-helix (Leu1333 and Leu1335) and H-loop (Ala1376, Gln1377 and Phe1378). With respect to the C-helix, we observed a correlation between the length of the hydrophobic lateral chain of Leu1333/1335 and the severity of the DNA condensation defects. When we reduced the length of the side chain at these positions, we observed an increase in severity of the phenotype. Such correlation could reflect a reduction or even loss of intramolecular interactions involving these positions, for example, by perturbing the position of the C-motif within the ATPase domain [52] (S5A Fig). Indeed, when we tested this notion with more dramatic changes that replaced both Leu1333 and Leu1335 for Ala, the resulting mutant was extremely sick and contracted its rDNA array. Likewise, mutations in the H-loop—in particular, at the positions encoding Gln1377 and its adjacent residues—also had major consequences for mitotic chromosome organization. Again, we observed a positive correlation between thermosensitive phenotypes associated with these alleles and the degree to which they affected chromosome condensation. In this case, the phenotypes are more severe when the typical polarity of this region is removed (S5B Fig). In both C-helix and H-loop, alanine mutations resulted in a contraction of the rDNA array and extremely sick mutants. Importantly, in vitro characterization of the purified condensin complexes carrying these mutations confirmed that their condensation defects are associated with decreased ATPase activity.

Interestingly, while the correlation between the compaction and ATPase defects is maintained in H-loop mutants, this is not the case with C-helix mutants, since strains carrying the Smc4-L1335A mutation are less thermosensitive than stains bearing the double L1333I-L1335A mutations, and yet the latter shows higher ATPase activity than the former. This loss of correlation likely indicates that the final phenotype of C-helix mutants may be the result of changes in other properties of condensin, for example, DNA binding. In particular, when Leu1335 residue is mutated, introduction of isoleucine at 1333 seem to act as a “second-site suppressor” in biochemical terms but not in genetic terms. This reverse relationship is consistent with the proposed catalytic mechanism of SMC proteins. Indeed, recent studies have shown that the ATPase activity of SMC proteins is a multistep process that involves structural changes upon binding of ATP to the SMC ATPase heads, followed by further changes in conformation after ATP hydrolysis and ADP release from the enzyme [9]. It is therefore likely that the L1333I “second-site suppressor” mutation relieved some of the structural constraints imposed by the L1335A mutation and improved ATPase activity by moving forward the enzymatic reaction past its initial stage (as observed in in vitro experiments in Fig 8A). However, the resulting enzyme might not be able to complete its catalytic cycle and may be trapped in a nonproductive conformation that would not allow effective compaction of chromatin in cells (thus explaining the more severe in vivo phenotype observed in Fig 3). This rationale predicts that the Smc4-L1333I-L1335A mutant has gained a novel biochemical property relative to the L1335A mutant and wild-type protein. Consistent with this view, we observed that mutation of both Leu1333 and Leu1335 significantly increased the affinity of the complex for ssDNA relative to the wild-type enzyme. A similar effect was also observed in the ATPase-dead mutant Smc4-K191M, suggesting that some mutations in the ATPase head domain might lock the enzyme in a specific configuration that promotes tight binding to DNA. Persistent binding to DNA might interfere significantly with condensin function in vivo and explain why the phenotype of Smc4-K191M and Smc4-L1333I-L1335A mutants is more severe than predicted solely by their defects in ATPase activity. It is interesting to note that Smc2 binds more avidly to ATP in condensin complexes containing the Smc4-K191M mutant, consistent with condensin adopting a specific configuration incompatible with viability in cells expressing this mutant. This interpretation is further supported by the observation that the C-motif of ABC-type ATPases contributes to ATP hydrolysis by different ATPase head domains in trans [55] and that loss of ATP hydrolysis in Smc4 may stabilize ATP molecules on Smc2 and result ultimately in the stabilization of a specific configuration of the ATPase head domains of Smc2 and Smc4 in condensin. The ATP-binding result we obtained with condensin complexes containing the Smc4-K191M mutant may appear surprising at first glance because a similar mutation in *B*. *subtilis* SMC resulted in complete abrogation of ATP binding [19]. However, bacterial SMC complexes are homodimeric, and mutations that prevent ATP binding on one SMC subunit will have similar effects on the other subunit. This will not be the case for SMC subunits of eukaryotic condensins, because they are heterodimeric, and it is conceivable, if not likely, that ATPase mutations in one SMC subunit could stabilize an ATP molecule on the other SMC member of the complex. Further structural work will be necessary to address this possibility and the nature of its functional consequences on condensin activity.

### Condensin ATP hydrolysis represents a limiting factor in mitotic chromosome organization

Our study reveals that cells contain an excess of condensin activity relative to the levels required for normal chromosome condensation during mitosis. In fact, protein down-regulation experiments show that Smc4 protein levels—and by extension, total condensin activity in cells—can be artificially reduced by more than 90%, and neither rDNA condensation nor viability is affected if the ATPase activity of the complex remains unperturbed [37]. This result is consistent with previous observations that cohesin activity can be reduced significantly without impairing its sister-chromatid cohesion activity in vivo [56]. Likewise, a recent study taking advantage of a rapid-degradation allele of *SMC2* has shown that metazoan condensins can be depleted to approximately 5% wild-type levels without major disruption of chromatin compaction in vivo [57]. It thus appears the notion that condensin ATPase activity is in excess in relationship to many of its cellular functions holds true over large evolutionary distances in eukaryotes (at least under optimal growth conditions). However, when condensin ATPase activity is fully compromised, chromosome condensation becomes completely defective in mitosis, and cells die, thus revealing different minimal thresholds of ATP activity required to execute different cellular functions. Comparative analysis of ATP hydrolysis rate in ATP-dead mutants (*smc4-K191M*) and other condensin ATPase mutants provides crucial insight into the contribution of ATP hydrolysis to the process of chromosome condensation. Indeed, mutation of the key lysine in the P-loop/Walker A motif is known to impair ATP binding [19], which explains the inviability of cells expressing this mutant. However, 2 of the mutants generated in this study (*smc4-L1335A* and *smc4-A1377S-Q1378A*) show an ATP hydrolysis rate similar to the ATPase-dead allele and yet are capable of sustaining cell viability. This suggests that the *smc4-K191M* mutant leads to a dominant (or semidominant) condensation defect because this specific mutation traps the enzyme in a deleterious configuration during the process of ATP hydrolysis. This hypothesis is consistent with the lethality we observed for transition state mutants like *smc4-E1352D/Q*, which is known to prevent disengagement of the head–head interactions [58]. These observations do not mean, however, that all constitutive lethal mutations that we identified in *SMC4* act in a dominant manner, since it appears likely that many mutants will be inviable as a result of a recessive defect in chromosome condensation and/or segregation. Beyond these mechanistic considerations, our observation that condensin ATP hydrolysis rate can be reduced significantly while maintaining viability suggests a model in which cells require minimal condensin ATPase activity to compact chromosomes under normal circumstances, independently of the total number of condensin molecules.

In closing, we note that the results of our study may have a number of implications for cancer treatment. Interrogation of the Catalogue of Somatic Mutations in Cancer (COSMIC) database [59] revealed that partial deletions of the ATPase domain of human Smc4 have been observed in a number of cancer patients (S6A and S6B Fig). We have introduced 1 cancer-specific deletion mutation at the homologous position of *SMC4* (i.e., Arg1384*) in diploid yeast and showed that this leads to lethality after sporulation (S6B Fig). A more modest deletion of the C-terminus of yeast Smc4 (i.e., Lys1410*; similar to a truncation made in *Geobacillus stearothermophilus* SMC [60]) did lead to a viable but sick yeast strain, suggesting that a partial loss of sequence at the C-terminal ATPase domain is possible and functionally impairs condensin activity. The COSMIC database also revealed that human *SMC4* is frequently overexpressed in cancers (Fig 6C and 6D). This, together with our observation that normal cells express an excess of condensin activity (relative to their normal need for chromosome condensation), suggests that targeting condensin with inhibitors of its ATPase activity might be an effective therapeutic approach to kill cancer cells without harming normal cells. Moreover, many cancer cells suffer from severe aneuploidy [61], a condition that we predict would impose an additional burden on the chromosome condensation machinery. In light of this, we propose that moderate inhibition of condensin ATPase activity might selectively kill aneuploid cells without affecting normal cells, thus providing a unique therapeutic window to target cancer cells. Consistent with this notion, it has been recently shown that Smc4 protein levels correlate with an aggressive phenotype in tumor cells, establishing a link between condensin levels and tumor progression in humans [62,63]. Assessment of condensin inhibition in a therapeutic setting will require the identification of inhibitors of the ATPase activity of this essential enzyme.

## Materials and methods

### Yeast strains and growth conditions

All yeast strains are derivatives of W303 (K699) [64], and their genotypes are summarized in S3 Table. Yeast culture conditions, sporulation, and dissection were performed by standard procedures. Synchronization of cultures in metaphase was performed using nocodazole (30 μg/ml for 150 min). To induce the degradation of Smc4-3xSTII-AID, cells were treated with indole-3-acetic acid (IAA) according to published procedures [65].

### Plasmid and *SMC4* mutant construction

Mutations were introduced in Smc4 subunit using QuikChange Multi Mutagenesis kit (Stratagene) in the plasmid YCplac111-*P*_*SMC4*_*-SMC4-Linker-3xStrepTagII*::*T*_*ADH1*_::*HIS3MX6*. Mutant alleles carry *smc4-F164A; smc4-[164–167]A; smc4-K165M; smc4-K165A; smc4-Y167C; smc4-K191M; smc4-R210A; smc4-R210M; smc4-R210K; smc4-R210D; smc4-R210P; smc4-R210A-Q211A; smc4-R210D-Q211E; smc4-Q302E; smc4-Q302K; smc4-Q302L; smc4-Q302D; smc4-S1324R; smc4-S1324N; smc4-K1328A; smc4-L1333A; smc4-L1335A; smc4-L1333A-L1335A; smc4-L1335G; smc4-L1333V-L1335A; smc4-L1333I-L1335A; smc4-P1346G; smc4-P1346T; smc4-P1346L; smc4-P1344G-P1346G; smc4-D1351A; smc4-D1351N; smc4-E1352Q; smc4-E1352D; smc4-D1358H; smc4-D1358N; smc4-D1358E; smc4-I1364N; smc4-V1365M; smc4-V1365F; smc4-Q1377A; smc4-Q1377R; smc4-A1376S-Q1377A; smc4-Q1377A- F1378A; smc4-L1383A; smc4-L1383I; smc4-L1383V; smc4-N1386H; smc4-N1386D; smc4-G1396Q; smc4-G1396P*. Mutant alleles of *SMC4* were introduced at the endogenous locus by transformation of digested plasmids, thus releasing a fragment containing *SMC4* ORF with its selection marker. The backbone of the plasmid was lost during the process. All the mutations were confirmed by sequencing the *SMC4* locus. For the overexpression of condensin subunits (wild-type and *smc4* ATPase alleles), *P*_*GAL1-10*_ or *P*_*GAL7*_-driven subunits (*P*_*GAL7*_*-SMC4-3xStrepTagII*, *P*_*GAL10*_*-SMC2*, *P*_*GAL1*_*-BRN1-3xHA-12xHIS*, *P*_*GAL10*_*-YCS4*, and *P*_*GAL1*_*-YCG1-3xFLAG-9xHIS*) were subcloned in tandem on 2 multicopy plasmids (*i*.*e*., *2μ TRP1 leu2-d P*_*GAL*_*-YCS4-YCG1 and 2μ URA3 leu2-d P*_*GAL*_*-SMC4-SMC2-BRN1*).

### FISH

Cells were fixed and harvested in 0.1M KPO_4_ buffer pH 6.4 containing 3.7% formaldehyde for 2 hours at 23 °C. The procedure to analyze the morphology of the rDNA locus is based on published data [40,66]. The probe was obtained and digoxigenin-labeled according to published procedures [37]. The digoxigenin-labeled DNA was detected using mouse anti-DIG antibody from Roche and FITC-conjugated goat anti-mouse IgG (Jackson Immunoresearch) and Alexa Fluor 488-conjugated rabbit anti-goat IgG antibodies (Jackson Immunoresearch). All 3 antibodies were diluted 1:250 using 10% horse serum prior to use. Nuclei were stained with propidium iodide (PI; Sigma) in 5 mg/ml of p-phenylenediamine (Sigma).

### Microscopy

Visualization of rDNA morphology was performed on DeltaVision microscope using the softWoRx software (Applied Precision). The microscope was equipped with a 100×/NA 1.4 Plan APO objective (Olympus) and a CoolSnap HQ2 camera (Photometrics). Images were acquired at 1 × 1 binning. Final images represent the maximum intensity projections of images taken at 0.2 μm intervals.

### Western blot

Whole cell extracts for immunoblot analysis were prepared using TCA/glass bead method [67]. Smc4 was separated by SDS-PAGE containing 8% acrylamide (BioRad). All gels were transferred using the iBlot system (Invitrogen). Membranes were probed with mouse monoclonal anti-StrepTagII (from Qiagen; at 1:1,000 dilution) and mouse monoclonal 22C5D8 (from Abcam; 1:10,000 dilution) in 2% milk and 1% BSA. The secondary antibody used was HRP-conjugated anti-mouse antibodies (10,000, Amersham/GE Healthcare). Protein–antibody conjugates were revealed by chemiluminescence (Western Lightning Plus-ECL; Perkin-Elmer).

### Protein purification

Proteins were overexpressed in yeast strain D1074 as described before, with few modifications [49]. Condensin complexes were overexpressed using p484 and variants of p490 containing the different *SMC4* ATPase alleles. Cells were grown in rich medium with 1% lactic acid and 3% glycerol, and protein overexpression was induced for 4 hours with galactose (2% final) in 4 liters of culture at 30 °C. Cells were resuspended in lysis buffer (10% glycerol, 500 mM NaCl, 10 mM Tris-HCl pH 8.0 supplemented with 10 μM E64, 1 mM AESBF, 10 μM pepstatin A), and the extract was prepared by grinding cells in liquid nitrogen in a SPEX CentriPrep 6850 Freezer Mill [68]. After sonication (3 pulses of 10 seconds at level 3 using a Misonix Sonicator 3000) and centrifugation, the extract was incubated with Ni-NTA agarose matrix (Quiagen), washed with 20 mM imidazole, and eluted with 500 mM imidazole. The elution from Ni-NTA is then applied on a Strep-Trap HP column (GE Healthcare). After a wash (50 mM Tris, 500 mM NaCl, 2 mM β-mercaptoethanol [β-ME], 1 mM EDTA. 0.7% Triton X-100, 10% Glycerol), condensin was eluted with buffer containing 20 mM desthiobiotin. Eluted fractions were concentrated by ultrafiltration using Vivaspin 20 (Sartorius) and then purified by size exclusion on Superose 6 10/300 (GE Healthcare) in FP buffer (200 mM NaCl, 50 mM Tris-HCl pH 8.0). Final fractions containing condensin were concentrated with Ultracell 100K (Millipore). Quantification of condensin subunit abundance was performed using Image J analysis of Coomassie-stained bands on SDS-polyacrylamide gels.

### ATP hydrolysis assay

ATPase assay was performed as previously published, with minor modifications [69]. Reaction mixture contained 10 nM HEPES pH 7.8, 120 mM NaCl, 12 mM Tris pH 7.8, 2 mM β-ME, 0.5 mM ATP, 100 μg/mL, and 1.5 μmol of condensin. The mixture was incubated at 30 °C for 1 hour, and the ADP production was measured with the ADP-Glo luminescence kit (Promega) with a microplate reader (BioTek).

### ATP-binding assay

Binding of ATP to yeast condensin was performed using the UV cross-linking procedure as previously described [70], with few modifications. First, 2.5 μmol of each condensin complex was prepared in 12.5 μl reaction buffer containing 10 mM HEPES (pH 8.0), 1 mM MgCl_2_, 1 mM β-ME, 45 nM [ϒ-P^32^]ATP (3,000 Ci/mmol). The mixtures were transferred to a parafilm wrap on ice and were exposed to UV radiation (254 nm) for 10 minutes to induce cross-link. After cross-linking, protein samples were separated by SDS-PAGE, and the radioactive signal was detected using a Typhoon FLA-9500 Bio-Image Analyzer. The ATP-bound signal detected was normalized to the protein signal detected in the Coomassie-stained gels.

### DNA-binding assay

Fluorescence anisotropy experiments were carried out at 10 nM 6-FAM ssDNA and variable concentrations of protein (0.001–6 μM). Anisotropy was measured 20 minutes after the incubation of the binding reaction at 30 °C in a microplate reader (BioTek) at room temperature (λ absorbance 485 nm and λ emission 525 nm). Normalized fluorescence anisotropy (“FA”) was calculated according to

$$FA\;=\;\frac{r_{n}-r_{o}}{r_{max}-r_{o}},$$

in which r_n_ is the anisotropy for each protein concentration, r_max_ is the anisotropy at the highest protein concentration, and r_0_ is the anisotropy without protein. To calculate the equilibrium association constant (Kd), the normalized fluorescence anisotropy was represented as a function of protein concentration, and a curve was fit with nonlinear regression algorithm using Prism. The DNA–protein binding constant was calculated in 3 experiments performed with the same batch of purified protein.

To analyze the DNA binding reversibility, we performed a DNA competition assay. First, 10 nM of 6-FAM DNA was incubated with 500 nM of each condensin complex for 20 minutes at 30 °C. Then, the complexes were incubated with increasing concentrations of unlabeled DNA (10 nM–3 μM ssDNA) for 20 more minutes at 30 °C. Anisotropy was measured in a microplate reader (BioTek) at room temperature (λ absorbance 485 nm and λ emission 525 nm). Normalized fluorescence anisotropy (FA) for the DNA competition assay was calculated according to

$$FA\;=\;\frac{r_{n}-r_{o}}{r_{max}-r_{o}},$$

in which r_n_ is the anisotropy for each DNA concentration, r_max_ is the anisotropy at the lowest ssDNA concentration, and r_0_ is the anisotropy with the same ration of 6-FAM ssDNA and not labeled DNA.

### qPCR analysis of rDNA copy number

*rDNA/RDN1* copy number in yeast strains was determined by qPCR analysis of the amplification signal obtained from genomic DNA isolated from a wild-type strain (D4107), the *25xRDN1* strain (D419), and the various condensin mutants generated in this study. The *RDN1* amplification signal was standardized relative to *SMC4* as a gene with a single copy number using the formula

$$RDN1\;amount\;=\;2^{\left(\Delta CtSMC4\;(strain-WT)-\Delta CtRDN1\;(strain-WT)\right)}$$

### Chromosome loss assay

The chromosome loss assay was performed by monitoring the loss of a chromosome III fragment (CFIII) carrying *HIS3* and *SUP11*, as previously described [41]. In brief, loss of CFIII leads to the formation of a red colony sector after growth on solid medium because of the failure of yeast cells to suppress the *ade2-1* mutation. The wild-type yeast strain and condensin mutants were grown in minimal liquid medium without histidine until mid/log phase at 23 °C and then incubated for 3 hours at 37°C. The cells were then diluted and plated in YPD medium without adenine supplementation. Cells were grown 4 days at 23 °C and then kept at 4 °C for 3 extra days to allow red color development. The number of colonies counted for each strain is as follows: *SMC4* (*N* = 4; 19,790 colonies), *smc4-22* (*N* = 5; 7,614), *smc4-L1335A* (*N* = 4; 13,697 colonies), *smc4-L1333I-L1335A* (*N* = 5; 12,021 colonies), *smc4-L1333I-L1335V* (*N* = 5; 7,129 colonies), *smc4-Q1377A* (*N* = 4; 15,323 colonies), and *smc4-A1376-Q1377A* (*N* = 3; 6,223 colonies).

### Chromatin spreads

Chromatin spreads were performed as described previously [43,71]. In brief, 5 OD_595nm_ equivalent of cells synchronized with nocodazole at 32 °C were washed in 1 ml of solution-1 (0.1 M KPO_4_ pH7.4, 0.5 mM MgCl_2_, 1.2 M sorbitol) and resuspended in solution-1 with 1 M DTT. Cells were spheroplasted by the addition of Zymolase (10 mg/ml) and incubated at 30 °C with rotation. Next, cells were washed with solution-2 (0.1 M MES pH 6.4, 0.5 mM MgCl_2_, 1 mM EDTA, 1 M sorbitol). Cells were applied to a glass slide, fixed, and lysed by the addition of a fixative solution (3.4% paraformaldehyde, 3.4% sucrose) and 1% NP-40. Immediately after, cells were spread using a plastic pipette rolled from one end of the slide to the other and then left to dry overnight. Next, slides were washed with 1× PBS and blocked with PBS + 10% BSA. Finally, slides were incubated with mouse monoclonal anti-Myc 9E10 antibody for 2 hours and Alexa Fluor 488-conjugated goat anti-mouse antibody for 2 hours. Nuclei were counterstained with DAPI (4’,6-diamidino-2-phenylindole). Images were acquired using a Zeiss LSM700 confocal microscope with an oil immersion 63× objective. Fluorescence intensity of Smc4 was measured using ImageJ software.

### Tridimensional representation and modeling

The modeling of Smc4 ATPase head has been carried out using the SWISS-MODEL website [72] and 1W1W [30] as a template. Regions encoding the Smc1 ATPase domain were aligned with the analogous regions of Smc4 using ClustalW algorithm with a gap opening penalty of 10 and a gap extension penalty of 0.1 [73]. Only the conserved regions were modeled by homology. All the representations have been performed using UCSF Chimera [74].

### Primary data

All numerical values, sample size, and statistics reported in Figs 4B, 4D, 5B, 5C, 6B, 7B, 8A, 8B, S3, S4A, S4B, S6C and S6D are described in S1 Data.

## Supporting information

S1 Fig Alignment of the sequence encoding the extreme carboxy-terminus of ABC-type transporters and SMC proteins. (Top) Model of Smc4 ATPase domain using *Sc*Smc1 (1W1W) [30] crystal structure as a template. The position of Glycine 1396 is shown in red. (Bottom) Carboxy-terminal sequences of the ATPase domains of SMC proteins and ABC-type transporters from various species. The sequence of the conserved H-loop is highlighted in green. ABC, ATP-binding cassette; *Sc*Smc1, *S*. *cerevisiae* Smc1; SMC, structural maintenance of chromosomes.
(TIF)

S2 Fig Similarity in the structures of *Sc*Smc1 and *Pf*Smc. A structural overlap analysis was conducted to depict the similarities in the crystal structures of SMC family members. Similarity value was obtained using Standard Protein BLAST. *Pf*Smc, *P*. *furiosus* Smc; *Sc*Smc1, *S*. *cerevisiae* Smc1; SMC, structural maintenance of chromosome.
(TIF)

S3 Fig Smc2 shows increased nucleotide retention after UV cross-linking in condensin complexes containing Smc4 ATPase mutants. (Top) The ATP-binding ability of Smc4, Smc4-K191M, Smc4-L1335A, and Smc4-Q1377A mutants were compared using equal amounts of protein. The two upper panels correspond to autoradiograms of [P^32^]ATP-labeled condensin subunits (long and short exposure), whereas the lower panel shows a Coomassie staining of the different condensin complexes used in this experiment. Data shown in this figure are from a representative experiment. (Bottom) The histogram on the left shows the relative ATP-binding activity of Smc4 with respect to that of Smc2 and Ycs4. Note that Smc2 and Ycs4 migrate at a similar position in SDS-PAGE and cannot be discriminated on standard gel, which is why the data is expressed relative to the combined signal of these two proteins. However, only Smc2 and Smc4 have the ability to bind ATP in the condensin complex. Error bars represent SD (*n* = 3 for all complexes). The histogram on the right reports the quantification of ATP bound to condensin complexes normalized for protein abundance (i.e., from the Coomassie-stained gel shown in the top panels). Error bars represent SD (*n* = 3 for all the complexes). See S1 Data for primary data. Note that the ATP-binding bands under Smc2/Ycs4 are likely to be chaperones, since they are known to bind ATP strongly, and they also associate with condensin under normal purification conditions [75]. Their increased abundance in Smc4-Q1377A- and Smc4-K191M-containing condensin likely reflects the mutated/partially distorted configuration of these complexes.
(TIF)

S4 Fig Condensin mutants bind ssDNA in a reversible manner. (A) DNA binding of Smc4, Smc4-K191M, and the H-loop and C-helix mutants was assessed as described in Fig 8B. The data were analyzed using nonlinear regression parameters and specific binding with Hill slope equation. Points and error bars indicate mean and standard error, respectively (*n* = 3). See S1 Data for primary data. (B) DNA competition assay of the condensin complexes used in (A) was analyzed using fluorescence anisotropy. Increasing amounts of unlabeled DNA (10 nM–3 μM) were incubated with preform 6-FAM ssDNA–condensin complexes. The data were analyzed using nonlinear regression parameters and one-phase decay equation. Points and error bars indicate mean and standard error, respectively (*n* = 3). See S1 Data for primary data. (C) Analysis of Smc4-Smc2-Ycs4-Ycg1-Brn1 subunit stoichiometry in mutant complexes after gel filtration, SDS-PAGE, and Coomassie staining. Quantification of the intensity (R.I.) of each band compared to their theoretical distribution, assuming condensin subunit intensity correlates with its molecular weight, and each is present at an equimolar concentration in the complex. 6-FAM, 6-carboxyfluorescein; R.I., relative intensity; ssDNA, single-stranded DNA.
(TIF)

S5 Fig In silico prediction of the structural effect of thermosensitive mutations. (A) Modeling of likely intramolecular interactions involving Leu1333 and Leu1335 within the C-helix of Smc4 and the impact of relevant mutations at those positions. Amino acid residues at position 1333 and 1335 are depicted in blue, while residues in the vicinity of these position (i.e., distance smaller than 5 Å) are depicted in red. (B) Model showing the hydrophobicity of the residues at position 1377 and 1378 in the H-loop of Smc4. Minimum hydrophobicity is depicted in blue and maximum in red.
(TIF)

S6 Fig Cancer-related mutations in human *SMC4*. (A) Analysis of the types of mutations found in *SMC4* gene in human cancers. The analysis was performed using the COSMIC database [59], and mutation types are represented in a pie chart format. (B) Cancer-related point mutations identified in *hSMC4* gene. The table on top highlights a subset of point mutations that were identified in *hSMC4*, how they affect hSmc4 protein sequence, their recurrence, and the corresponding residue changes in yeast Smc4. Note that N447H and S1056L mutations are not localized in evolutionarily conserved regions of *hSMC4* sequence, while R1252 affects the conserved ATPase head domain of the protein. The lower part of this panel shows the growth properties of spores carrying smc4-R1384* and smc4-K1410* alleles after dissection of heterozygous diploid strains. The smc4-R1384* truncation allele corresponds to the R1252* mutation in *hSMC4*, whereas the smc4-K1410* truncation corresponds to a C-terminal deletion allele introduced in *G*. *stearothermophilus* SMC [60]. (C) Overexpression profile of *SMC4* in various types of human cancers. Data are from the COSMIC database. See S1 Data for primary data. (D) Frequencies of point mutations in *SMC4* relative to *SMC4* gene overexpression in human cancers. See S1 Data for primary data. COSMIC, Catalogue of Somatic Mutations in Cancer; hSmc4, human Smc4.
(TIF)

S1 Data Primary data. Table reporting measured and calculated values for individual experimental data shown in Figs 4B, 4D, 5B, 5C, 6B, 7B, 8A, 8B, S3, S4A, S6B, S6C and S6D. Each experiment is described on a different sheet.
(XLSX)

S1 Table Chromosome morphology in *SMC4*, *smc4-22*, *smc4-7A*, and ATPase mutants. Statistical significance was calculated using one-way ANOVA with Dunnett’s post hoc test (using wild-type *SMC4* as control group). Star symbols signify the following *p* values: * *p* < 0.05, ** *p* < 0.01, *** *p* < 0.001, and ****p < 0.0001. N.s. indicates no significant difference, whereas a dash (–) symbol indicates not tested or not relevant.
(XLSX)

S2 Table Summary of the phenotypes associated with *smc4* ATPase mutations. The following abbreviations and symbols signify: +++, very severe defect / ++, moderately severe defect / +, weak defect / -, no detectable phenotype (or not tested) / c.s., cold-sensitive / n.r., not relevant / Y, Yes / N, No.
(XLSX)

S3 Table Yeast strains used in this study. Only the relevant genotype differences are shown.
(XLSX)

## Abbreviations

ABC: ATP-binding cassette
CAP-H: chromosome-associated protein H
CFIII: chromosome III fragment
COSMIC: Catalogue of Somatic Mutations in Cancer
CTF: chromosome transmission fidelity
dsDNA: double-stranded DNA
FISH: fluorescence in situ hybridization
IAA: indole-3-acetic acid
*Pf*Smc: *Pyrococcus furiosus* Smc
PI: propidium iodine
rDNA: ribosomal DNA
*Sc*Smc1: *Saccharomyces cerevisiae* Smc1
SMC: structural maintenance of chromosomes
ssDNA: single-stranded DNA
