# Supplementary figures and images for: Condensin ATPase motifs contribute differentially to the maintenance of chromosome morphology and genome stability
Source: PLoS Biol. 2018 Jun 27;16(6):e2003980. doi: 10.1371/journal.pbio.2003980 (PMC6039025; doi:10.1371/journal.pbio.2003980)

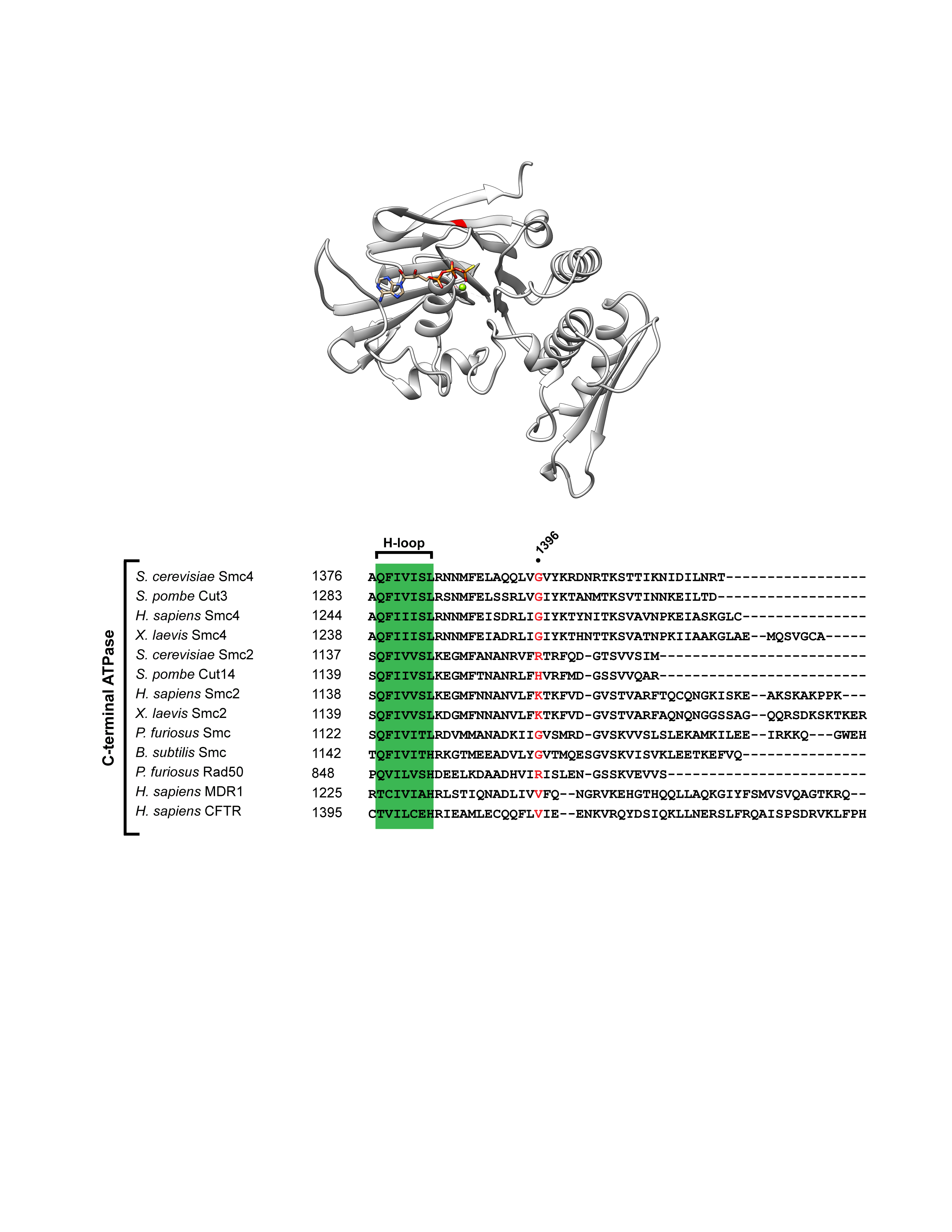

Supplement: S1 Fig — (Top) Model of Smc4 ATPase domain using ScSmc1 (1W1W) [30] crystal structure as a template. The position of Glycine 1396 is shown in red. (Bottom) Carboxy-terminal sequences of the ATPase domains of SMC proteins and ABC-type transporters from various species. The sequence of the conserved H-loop is highlighted in green. ABC, ATP-binding cassette; ScSmc1, S. cerevisiae Smc1; SMC, structural maintenance of chromosomes. (TIF) [file pbio.2003980.s001.tif]

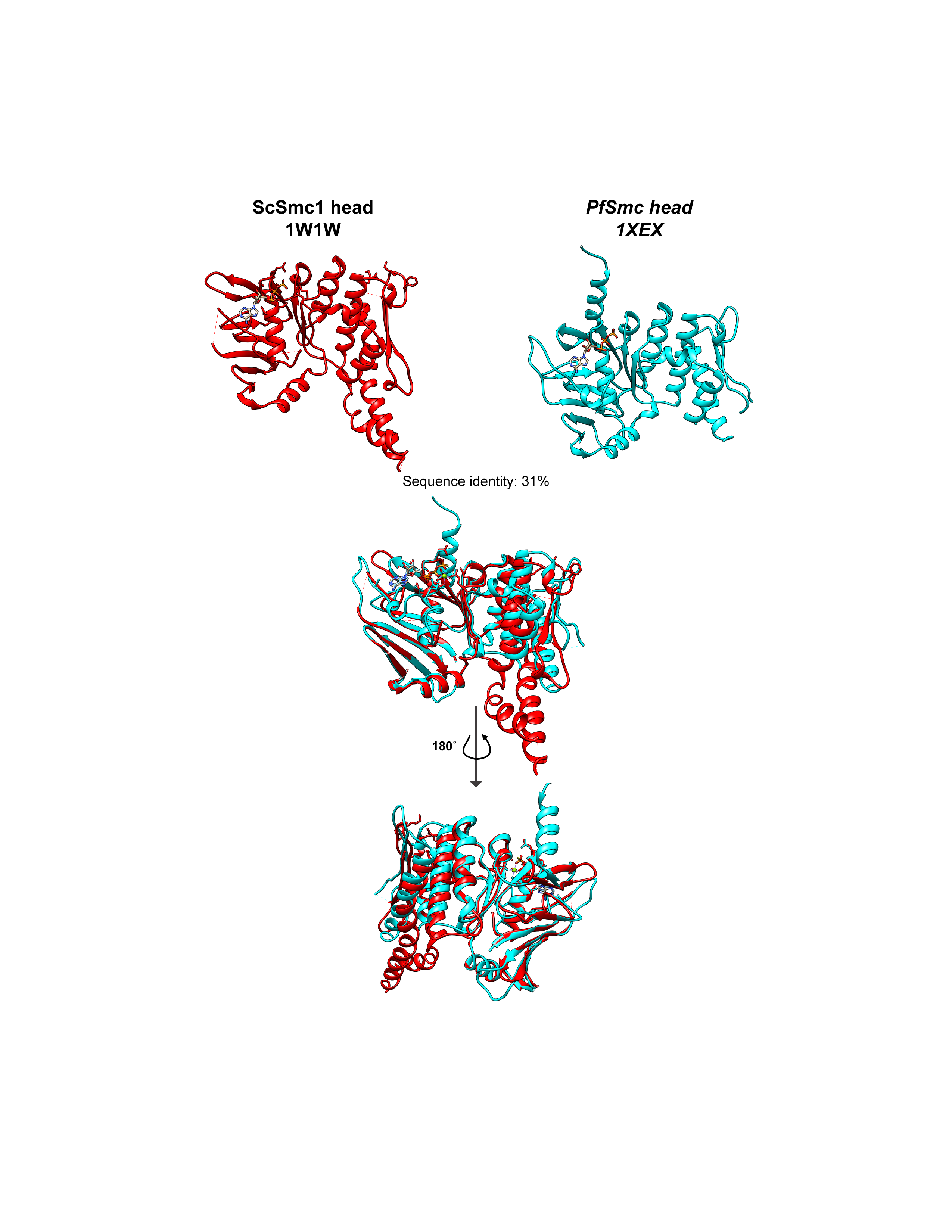

Supplement: S2 Fig — A structural overlap analysis was conducted to depict the similarities in the crystal structures of SMC family members. Similarity value was obtained using Standard Protein BLAST. PfSmc, P. furiosus Smc; ScSmc1, S. cerevisiae Smc1; SMC, structural maintenance of chromosome. (TIF) [file pbio.2003980.s002.tif]

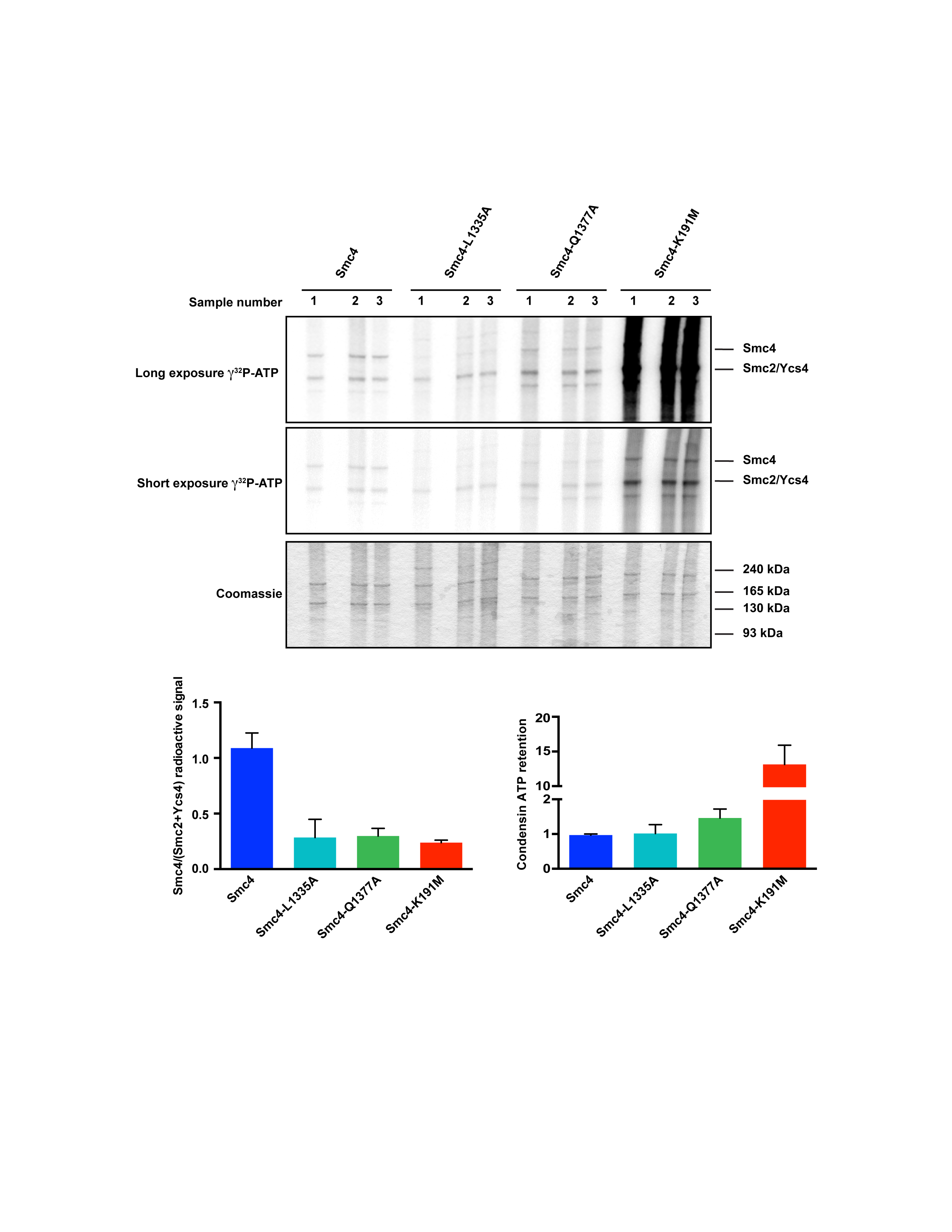

Supplement: S3 Fig — (Top) The ATP-binding ability of Smc4, Smc4-K191M, Smc4-L1335A, and Smc4-Q1377A mutants were compared using equal amounts of protein. The two upper panels correspond to autoradiograms of [P32]ATP-labeled condensin subunits (long and short exposure), whereas the lower panel shows a Coomassie staining of the different condensin complexes used in this experiment. Data shown in this figure are from a representative experiment. (Bottom) The histogram on the left shows the relative ATP-binding activity of Smc4 with respect to that of Smc2 and Ycs4. Note that Smc2 and Ycs4 migrate at a similar position in SDS-PAGE and cannot be discriminated on standard gel, which is why the data is expressed relative to the combined signal of these two proteins. However, only Smc2 and Smc4 have the ability to bind ATP in the condensin complex. Error bars represent SD (n = 3 for all complexes). The histogram on the right reports the quantification of ATP bound to condensin complexes normalized for protein abundance (i.e., from the Coomassie-stained gel shown in the top panels). Error bars represent SD (n = 3 for all the complexes). See S1 Data for primary data. Note that the ATP-binding bands under Smc2/Ycs4 are likely to be chaperones, since they are known to bind ATP strongly, and they also associate with condensin under normal purification conditions [75]. Their increased abundance in Smc4-Q1377A- and Smc4-K191M-containing condensin likely reflects the mutated/partially distorted configuration of these complexes. (TIF) [file pbio.2003980.s003.tif]

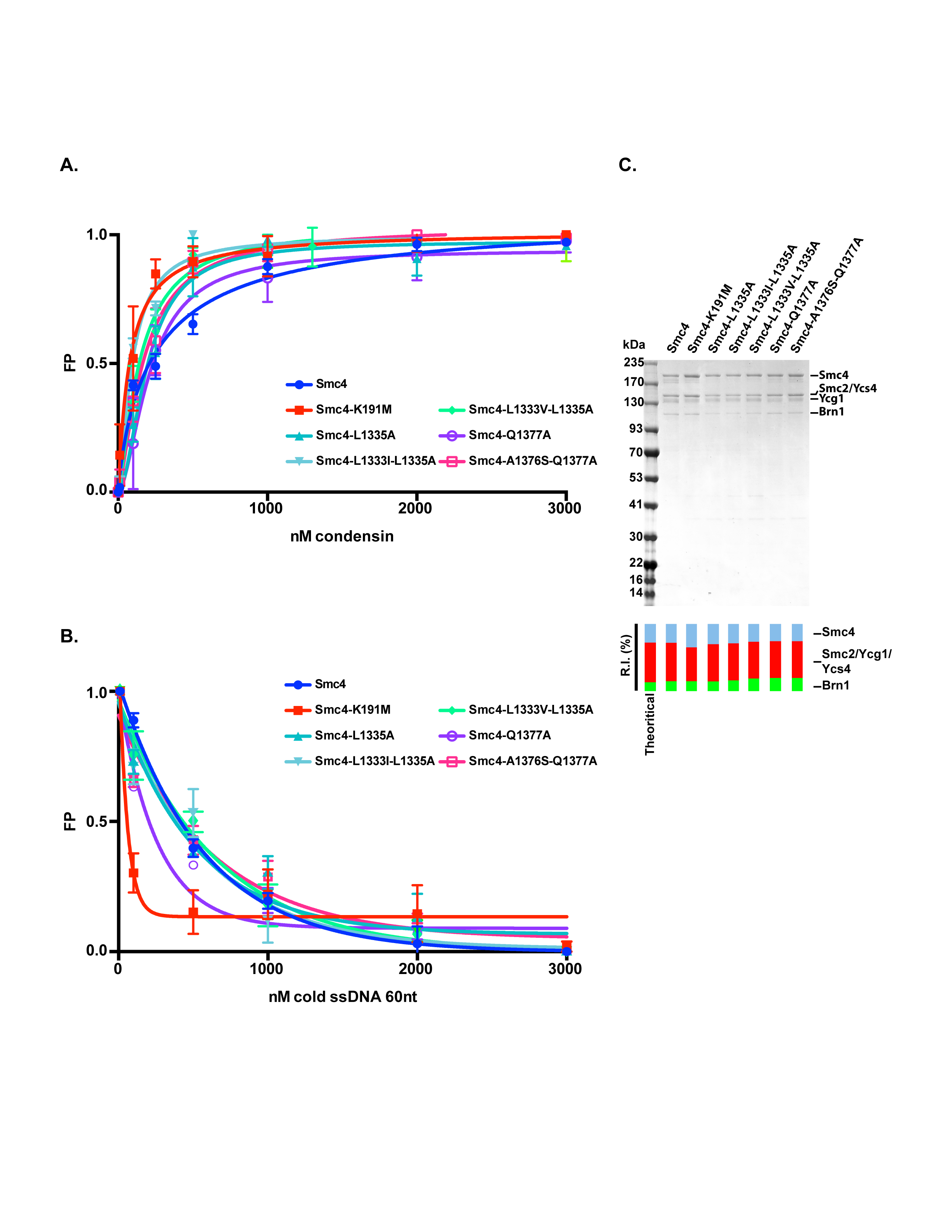

Supplement: S4 Fig — (A) DNA binding of Smc4, Smc4-K191M, and the H-loop and C-helix mutants was assessed as described in Fig 8B. The data were analyzed using nonlinear regression parameters and specific binding with Hill slope equation. Points and error bars indicate mean and standard error, respectively (n = 3). See S1 Data for primary data. (B) DNA competition assay of the condensin complexes used in (A) was analyzed using fluorescence anisotropy. Increasing amounts of unlabeled DNA (10 nM–3 μM) were incubated with preform 6-FAM ssDNA–condensin complexes. The data were analyzed using nonlinear regression parameters and one-phase decay equation. Points and error bars indicate mean and standard error, respectively (n = 3). See S1 Data for primary data. (C) Analysis of Smc4-Smc2-Ycs4-Ycg1-Brn1 subunit stoichiometry in mutant complexes after gel filtration, SDS-PAGE, and Coomassie staining. Quantification of the intensity (R.I.) of each band compared to their theoretical distribution, assuming condensin subunit intensity correlates with its molecular weight, and each is present at an equimolar concentration in the complex. 6-FAM, 6-carboxyfluorescein; R.I., relative intensity; ssDNA, single-stranded DNA. (TIF) [file pbio.2003980.s004.tif]

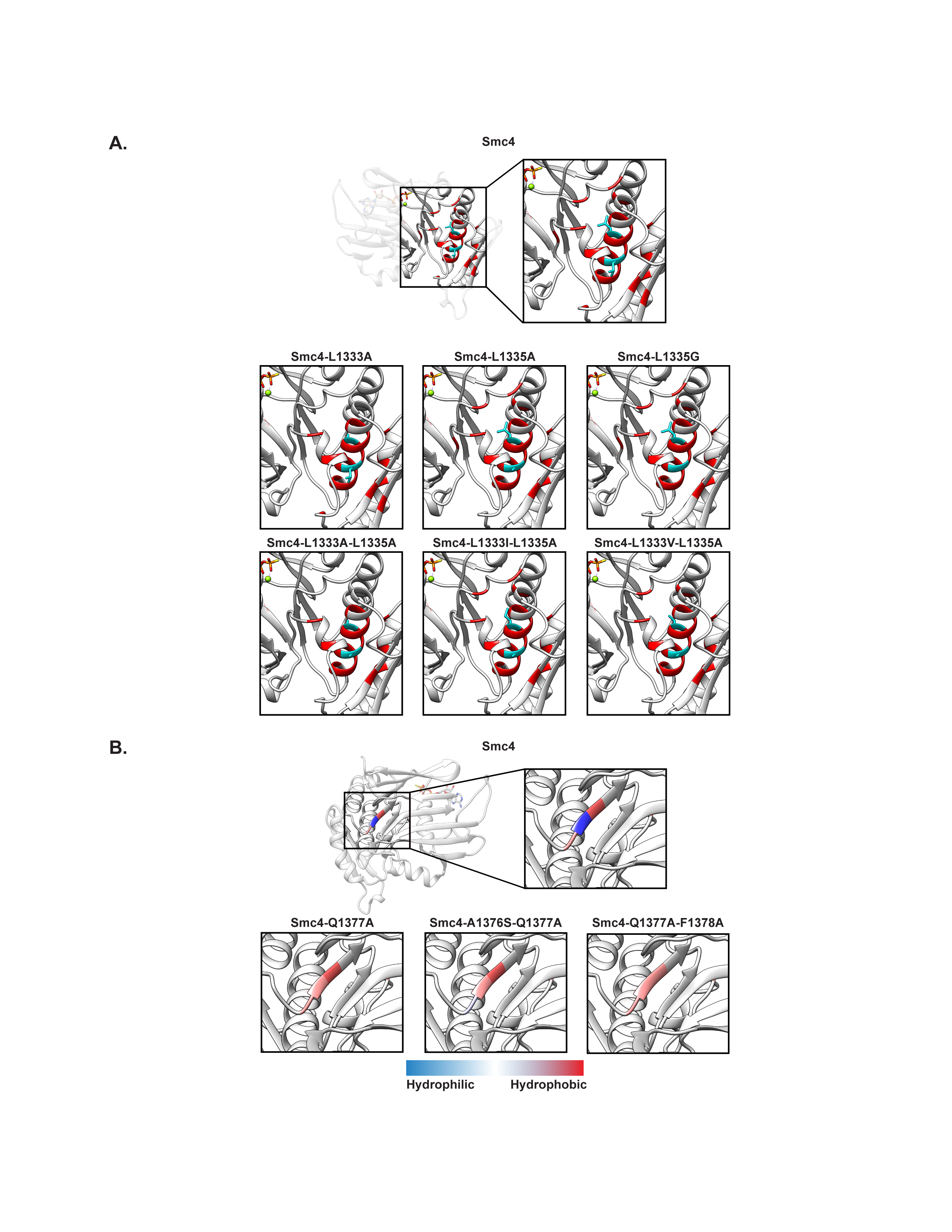

Supplement: S5 Fig — (A) Modeling of likely intramolecular interactions involving Leu1333 and Leu1335 within the C-helix of Smc4 and the impact of relevant mutations at those positions. Amino acid residues at position 1333 and 1335 are depicted in blue, while residues in the vicinity of these position (i.e., distance smaller than 5 Å) are depicted in red. (B) Model showing the hydrophobicity of the residues at position 1377 and 1378 in the H-loop of Smc4. Minimum hydrophobicity is depicted in blue and maximum in red. (TIF) [file pbio.2003980.s005.tif]

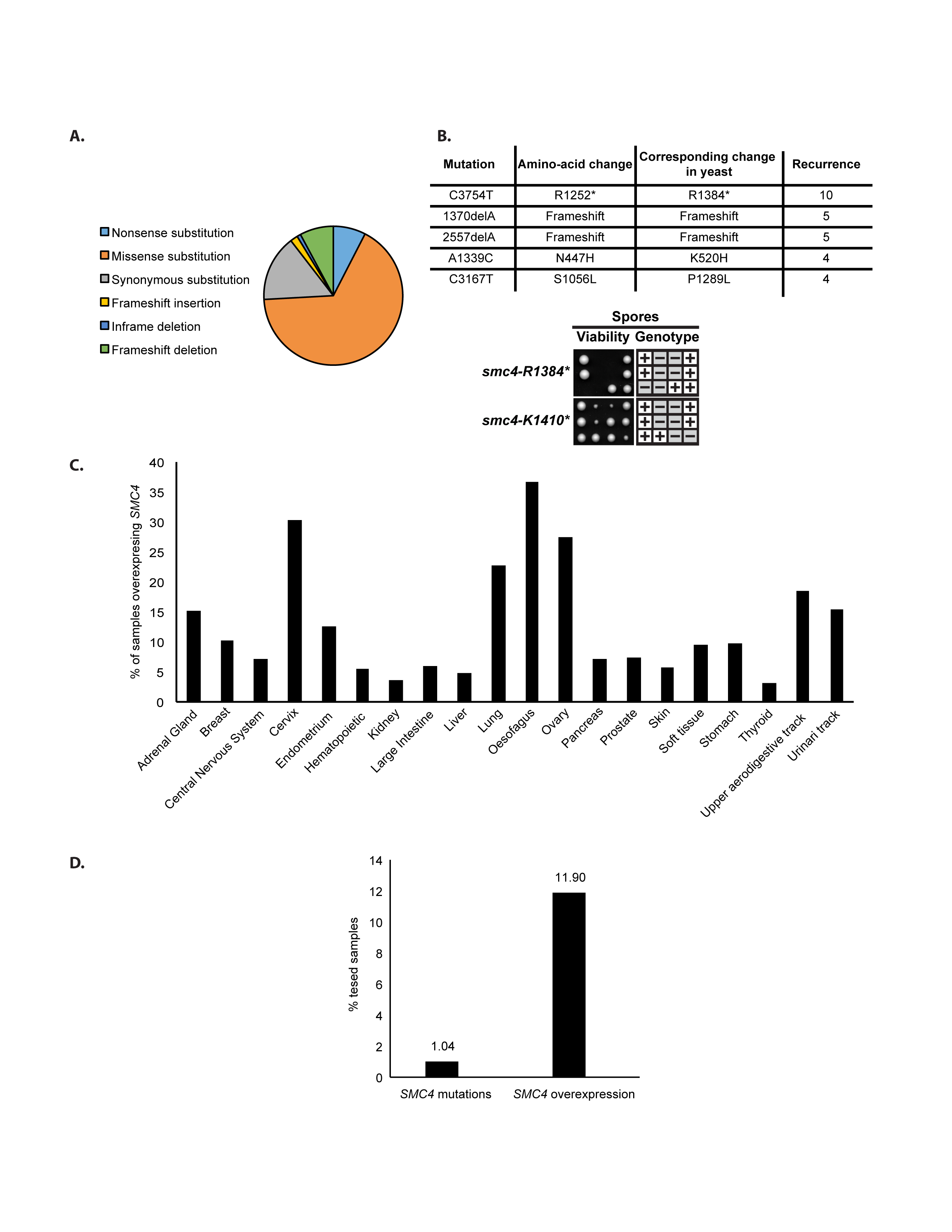

Supplement: S6 Fig — (A) Analysis of the types of mutations found in SMC4 gene in human cancers. The analysis was performed using the COSMIC database [59], and mutation types are represented in a pie chart format. (B) Cancer-related point mutations identified in hSMC4 gene. The table on top highlights a subset of point mutations that were identified in hSMC4, how they affect hSmc4 protein sequence, their recurrence, and the corresponding residue changes in yeast Smc4. Note that N447H and S1056L mutations are not localized in evolutionarily conserved regions of hSMC4 sequence, while R1252 affects the conserved ATPase head domain of the protein. The lower part of this panel shows the growth properties of spores carrying smc4-R1384* and smc4-K1410* alleles after dissection of heterozygous diploid strains. The smc4-R1384* truncation allele corresponds to the R1252* mutation in hSMC4, whereas the smc4-K1410* truncation corresponds to a C-terminal deletion allele introduced in G. stearothermophilus SMC [60]. (C) Overexpression profile of SMC4 in various types of human cancers. Data are from the COSMIC database. See S1 Data for primary data. (D) Frequencies of point mutations in SMC4 relative to SMC4 gene overexpression in human cancers. See S1 Data for primary data. COSMIC, Catalogue of Somatic Mutations in Cancer; hSmc4, human Smc4. (TIF) [file pbio.2003980.s006.tif]
